# Supplementary material for: Inferring causal direction between two traits in the presence of horizontal pleiotropy with GWAS summary data
Source: PLoS Genet. 2020 Nov 2;16(11):e1009105. doi: 10.1371/journal.pgen.1009105 (PMC7660933; doi:10.1371/journal.pgen.1009105)
Supplement: S1 Text — (PDF) [file pgen.1009105.s001.pdf]

S1 Text: Asymptotic Covariance Matrix of $\boldsymbol{r}_{Xg}$ and $\boldsymbol{r}_{Yg}$

In the main text when we use multiple correlated SNPs to estimate correlation ratio, we use the asymptotic distributions of $\boldsymbol{r}_{Xg}$ and $\boldsymbol{r}_{Yg}$:

$$\sqrt{n_X} \cdot (\boldsymbol{r}_{Xg} - \boldsymbol{\rho}_{Xg}) \to N(0, \mathbf{V}_X), \quad \sqrt{n_Y} \cdot (\boldsymbol{r}_{Yg} - \boldsymbol{\rho}_{Yg}) \to N(0, \mathbf{V}_Y) \tag{1}$$

Here we show the calculation of asymptotic covariance matrix $\mathbf{V}_X$, and $\mathbf{V}_Y$ can be calculated similarly.

Suppose we have m SNPs denoted by $g_1, g_2, \cdots, g_m$ with trait X. From GWAS summary statistics, we can get sample correlations $\boldsymbol{r}_{Xg} \in \mathbb{R}^{m \times 1}$ between g's and X. From a reference panel like 1000-Genomes Project with n individuals, we get individual level genotype data for g_i as $\boldsymbol{g}_i = (g_{i1}, g_{i2}, \cdots, g_{in})^T$, $i = 1, 2, \cdots, m$. Because we only care about correlations, we can assume g's and X are standardized to have mean 0 and variance 1 in both GWAS data and reference panel. Denote $\mathbf{G} = (\boldsymbol{g}_1, \boldsymbol{g}_2, \cdots \boldsymbol{g}_m) \in \mathbb{R}^{n \times m}$. We can estimate the correlation matrix of $g_1, g_2, \cdots, g_m$, denoted by $\boldsymbol{\Sigma} = \mathbf{G}^T \mathbf{G}/n \in \mathbb{R}^{m \times m}$, as an approximation of sample correlation matrix of $g_1, g_2, \cdots, g_m$ calculated from the GWAS individual level data of X. So we can get the sample correlation matrix $\hat{\mathbf{P}} \in \mathbb{R}^{(m+1)\times(m+1)}$ of $x = (g_1, g_2, \cdots, g_m, X)^T$:

$$\hat{\mathbf{P}} = \begin{pmatrix} \boldsymbol{\Sigma} & \boldsymbol{r}_{Xg} \\ \boldsymbol{r}_{Xg}^T & 1 \end{pmatrix} \tag{2}$$

And denote $\mathbf{P} \in \mathbb{R}^{(m+1)\times(m+1)}$ is the true correlation matrix of $g_1, g_2, \cdots, g_m$ and X. Then we can apply Theorem 2 from [1] to get:

$$\sqrt{n_X} \text{vec}(\hat{\mathbf{P}} - \mathbf{P}) \xrightarrow{D} N(0, \mathbf{A}) \tag{3}$$

Here $\mathbf{A} = [\mathbf{I} - \mathbf{M}_s(\mathbf{I} \otimes \mathbf{P})\mathbf{M}_d](\boldsymbol{\Lambda}^{-1/2} \otimes \boldsymbol{\Lambda}^{-1/2})\mathbf{V}(\boldsymbol{\Lambda}^{-1/2} \otimes \boldsymbol{\Lambda}^{-1/2})[\mathbf{I} - \mathbf{M}_d(\mathbf{I} \otimes \mathbf{P})\mathbf{M}_s]$. Here "$\otimes$" is Kronecker product of two matrices.

Matrices $\mathbf{M_s}, \mathbf{M_d} \in \mathbb{R}^{(m+1)^2 \times (m+1)^2}$ can be calculated from equations (2.9) and (2.13) from [1]. We can plug in $\hat{\mathbf{P}}$ to replace $\mathbf{P}$. And because we assume g's and X are standardized to have variance 1, $\boldsymbol{\Lambda}$ is identity matrix. Then we need to calculate $\mathbf{V}$.

Using equation (3.6) in [1], because we standardized g's and X, the covariance matrix is the same as correlation matrix, we have:

$$\mathbf{V} = E[(x-\mu)(x-\mu)^T \otimes (x-\mu)(x-\mu)^T] - (\text{vec}\mathbf{P})(\text{vec}\mathbf{P})^T \tag{4}$$

Here $x = (g_1, g_2, \cdots, g_m, X)^T$ and $\mu = E(x)$. So we need $E[(x-\mu)(x-\mu)^T \otimes (x-\mu)(x-\mu)^T]$.

For n individuals in the reference panel, denote their unobserved X values as $\boldsymbol{X} = (X_1, X_2, \cdots, X_n)^T$, again $\boldsymbol{X}$ is standardized with mean 0 and variance 1. We fit a joint linear model of X on $g_1, g_2, \cdots, g_m$:

$$X_i = \beta_1 \cdot g_{1i} + \beta_2 \cdot g_{2i} + \cdots + \beta_m \cdot g_{mi} + \varepsilon_i \tag{5}$$

Here $\varepsilon_i \sim N(0, \sigma^2)$ is random error. We have $\hat{\boldsymbol{\beta}} = (\mathbf{G}^T\mathbf{G})^{-1}\mathbf{G}\boldsymbol{X} = (\mathbf{G}^T\mathbf{G}/n)^{-1}\mathbf{G}\boldsymbol{X}/n$. Here $\mathbf{G}^T\mathbf{G}/n = \boldsymbol{\Sigma}$, and $\mathbf{G}\boldsymbol{X}/n$ are correlations of g's with X which could be replaced with $\boldsymbol{r}_{Xg}$ from GWAS summary statistics. So we get $\hat{\boldsymbol{\beta}} = \boldsymbol{\Sigma}^{-1}\boldsymbol{r}_{Xg}$, and $\hat{\sigma}^2 = ||\boldsymbol{X} - \mathbf{G}\hat{\boldsymbol{\beta}}||^2/n = 1 - \boldsymbol{r}_{Xg}^T\boldsymbol{\Sigma}^{-1}\boldsymbol{r}_{Xg}$. So we can approximate $X_i = \hat{\beta}_1 \cdot g_{1i} + \hat{\beta}_2 \cdot g_{2i} + \cdots + \hat{\beta}_{mi} \cdot g_m + \hat{\varepsilon}_i$ with independently generated $\hat{\varepsilon}_i \sim N(0, \hat{\sigma}^2)$. With this representation of X_i in the reference panel, we can calculate the sample version of $E[(x-\mu)(x-\mu)^T \otimes (x-\mu)(x-\mu)^T]$ as its estimate. Thus, we get $\mathbf{V}$.

In summary we can estimate $\mathbf{A}$ in (3) which is the asymptotic covariance matrix of $\hat{\mathbf{P}}$, by extracting elements in $\mathbf{vec}(\hat{\mathbf{P}})$ corresponding to $\boldsymbol{r}_{Xg}$, we can get the asymptotic covariance matrix $\mathbf{V}_X$ of $\boldsymbol{r}_{Xg}$.

References

[1] Neudecker, H., & Wesselman, A. (1990). The asymptotic variance matrix of the sample correlation matrix. *Linear Algebra and Its Applications, 127*(C), 589-599.
