# Supplementary material for: Inferring causal direction between two traits in the presence of horizontal pleiotropy with GWAS summary data
Source: PLoS Genet. 2020 Nov 2;16(11):e1009105. doi: 10.1371/journal.pgen.1009105 (PMC7660933; doi:10.1371/journal.pgen.1009105)
Supplement: S2 Text — (PDF) [file pgen.1009105.s002.pdf]

S2 Text: Details of CD-Egger and CD-GLS

Distribution of $\boldsymbol{b}_{Yg}$

In the main text, when there are direct effects from g's to Y, we got

$$\rho_{Yg} = (\alpha_{Yg} + \beta_{YX} \cdot \beta_{Xg}) \cdot \frac{\sqrt{var(g)}}{\sqrt{var(Y)}} = \alpha_{Yg} \cdot \frac{\sqrt{var(g)}}{\sqrt{var(Y)}} + K_{YX} \cdot \rho_{Xg} = b_{Yg} + K_{YX} \cdot \rho_{Xg}. \quad (1)$$

Here α_{Yg}'s are the direct effects of g's to Y in the *marginal model*. g's are standardized to have mean 0 and variance 1, and the covariance matrix of g's is $\mathbf{\Sigma}$. If we assume the following joint model:

$$Y = \beta_{Y0} + \beta_{YX} \cdot X + \alpha^{(0)}_{Yg_1} \cdot g_1 + \cdots + \alpha^{(0)}_{Yg_m} \cdot g_m + U + \varepsilon_Y \quad (2)$$

Here $\alpha^{(0)}_{Yg_i}$'s are direct effects in the *joint model*, and assume $\alpha^{(0)}_{Yg_i}$'s are i.i.d. $N(b_0, \sigma_0^2)$. Then

$$\begin{aligned} \boldsymbol{\rho}_{Yg} =& \frac{1}{\sqrt{var(Y)}} \begin{pmatrix} cov(Y,g_1) \\ \vdots \\ cov(Y,g_m) \end{pmatrix} = \frac{1}{\sqrt{var(Y)}} \left(\beta_{YX} \cdot \begin{pmatrix} cov(X,g_1) \\ \vdots \\ cov(X,g_m) \end{pmatrix} + \mathbf{\Sigma} \begin{pmatrix} \alpha^{(0)}_{Yg_1} \\ \vdots \\ \alpha^{(0)}_{Yg_m} \end{pmatrix} \right) \\ =& \beta_{YX} \frac{\sqrt{var(X)}}{\sqrt{var(Y)}} \begin{pmatrix} \frac{cov(X,g_1)}{\sqrt{var(X)}} \\ \vdots \\ \frac{cov(X,g_m)}{\sqrt{var(X)}} \end{pmatrix} + \frac{\mathbf{\Sigma}}{\sqrt{var(Y)}} \begin{pmatrix} \alpha^{(0)}_{Yg_1} \\ \vdots \\ \alpha^{(0)}_{Yg_m} \end{pmatrix} \\ =& K_{YX} \cdot \boldsymbol{\rho}_{Xg} + \frac{\mathbf{\Sigma}}{\sqrt{var(Y)}} \begin{pmatrix} \alpha^{(0)}_{Yg_1} \\ \vdots \\ \alpha^{(0)}_{Yg_m} \end{pmatrix} \end{aligned} \quad (3)$$

So we have

$$\boldsymbol{b}_{Yg} = \frac{\mathbf{\Sigma}}{\sqrt{var(Y)}} \begin{pmatrix} \alpha^{(0)}_{Yg_1} \\ \vdots \\ \alpha^{(0)}_{Yg_m} \end{pmatrix} \sim N\left(\frac{b_0 \cdot \mathbf{\Sigma 1}}{\sqrt{var(Y)}}, \frac{\sigma_0^2 \mathbf{\Sigma}^2}{var(Y)} \right) \quad (4)$$

CD-Egger: Estimation of (b_0, K_{YX}) and σ_0^2

For CD-Egger, we have:

$$\boldsymbol{r}_{Yg} = b_0 \cdot \boldsymbol{v} + K_{YX} \cdot \boldsymbol{r}_{Xg} + \boldsymbol{\varepsilon}, \boldsymbol{\varepsilon} \sim N(0, \frac{\mathbf{V}_{Yg}}{n_Y} + \sigma_0^2 \mathbf{\Sigma}^2 = \mathbf{V}_{YX}) \quad (5)$$

We use a iterative algorithm to estimate (b_0, K_{YX}) and σ_0^2. Set initial $\sigma_0^2 = 0$.

Step (1): Given σ_0^2, we can get $\mathbf{V}_{YX} = \frac{\mathbf{V}_{Yg}}{n_Y} + \sigma_0^2 \mathbf{\Sigma}^2$. Then estimating (b_0, K_{YX}) is a Generalize Least Square problem with design matrix $\mathbf{X}$ and covariance matrix of errors $\mathbf{\Omega}$:

$$\begin{aligned} \mathbf{X} &= \begin{pmatrix} \boldsymbol{v} & \boldsymbol{r}_{Xg} \end{pmatrix} = \begin{pmatrix} v_1 & r_{Xg_1} \\ \vdots & \vdots \\ v_m & r_{Xg_m} \end{pmatrix} \\ \mathbf{\Omega} &= \mathbf{V}_{YX} \end{aligned} \quad (6)$$

So we can get the estimation $(\hat{b}_0, \hat{K}_{YX})$ and their covariance matrix:

$$\begin{aligned} \begin{pmatrix} \hat{b}_0 \\ \hat{K}_{YX} \end{pmatrix} &= (\mathbf{X}^T \mathbf{\Omega}^{-1} \mathbf{X})^{-1} \mathbf{X}^T \mathbf{\Omega}^{-1} \boldsymbol{r}_{Yg} \\ cov \begin{pmatrix} \hat{b}_0 \\ \hat{K}_{YX} \end{pmatrix} &= (\mathbf{X}^T \mathbf{\Omega}^{-1} \mathbf{X})^{-1} \end{aligned} \tag{7}$$

Step (2): Given $(\hat{b}_0, \hat{K}_{YX})$, plug them in equation (5), denoting

$$\boldsymbol{x}_{YX} = \boldsymbol{r}_{Yg} - \hat{b}_0 \cdot \boldsymbol{v} - \hat{K}_{YX} \cdot \boldsymbol{r}_{Xg} \tag{8}$$

We get

$$\mathbf{\Sigma}^{-1} \boldsymbol{x}_{YX} \sim N(0, \frac{\mathbf{\Sigma}^{-1} \mathbf{V}_{Yg} \mathbf{\Sigma}^{-1}}{n_Y} + \sigma_0^2 \mathbf{I}) \tag{9}$$

Do the eigenvalue decomposition of

$$\frac{\mathbf{\Sigma}^{-1} \mathbf{V}_{Yg} \mathbf{\Sigma}^{-1}}{n_Y} = \mathbf{Q}^T \begin{pmatrix} \lambda_1 & \cdots & 0 \\ \vdots & \ddots & \vdots \\ 0 & \cdots & \lambda_m \end{pmatrix} \mathbf{Q} \tag{10}$$

Here $\mathbf{Q}$ is orthonormal, $\lambda_1, \cdots, \lambda_m$ are eigen-values. Denote $\mathbf{Q}\mathbf{\Sigma}^{-1} \boldsymbol{x}_{YX} = \boldsymbol{x}$, we get:

$$\boldsymbol{x} \sim N(0, \begin{pmatrix} \lambda_1 + \sigma_0^2 & \cdots & 0 \\ \vdots & \ddots & \vdots \\ 0 & \cdots & \lambda_m + \sigma_0^2 \end{pmatrix}) \tag{11}$$

Then the log-likelihood function

$$l = -\frac{1}{2}(\frac{x_1^2}{\lambda_1 + \sigma_0^2} + \cdots + \frac{x_m^2}{\lambda_m + \sigma_0^2}) - \frac{1}{2}(\ln(\lambda_1 + \sigma_0^2) + \cdots + \ln(\lambda_m + \sigma_0^2)) + Constant \tag{12}$$

To get the MLE of σ_0^2, it is equivalent to get root of

$$\frac{x_1^2}{(\lambda_1 + \sigma_0^2)^2} + \cdots + \frac{x_m^2}{(\lambda_m + \sigma_0^2)^2} = \frac{1}{\lambda_1 + \sigma_0^2} + \cdots + \frac{1}{\lambda_1 + \sigma_0^2} \tag{13}$$

We can use the bisection method to search root for (18) and get the root, i.e., MLE of σ_0^2, denoted as $\hat{\sigma}_0^2$.

Step (3): Update $\mathbf{V}_{YX}$ with $\hat{\sigma}_0^2$, then repeat Step (1) and Step (2) iteratively until convergence, to get the final estimations $(\hat{b}_0, \hat{K}_{YX})$ and $\hat{\sigma}_0^2$. And we can get $se(\hat{b}_0)$ and $se(\hat{K}_{YX})$ from (7).

CD-GLS: Derivation and Estimation of (b_0, K_{YX}) and σ_0^2

In **CD-Egger** a possible downside is we ignore the variability in $\boldsymbol{r}_{Xg}$ while account for that of $\boldsymbol{r}_{Yg}$. We propose following method **Causal Direction-GLS**, and **CD-GLS** for short, to take into consider of both variation in $\boldsymbol{r}_{Xg}$ and $\boldsymbol{r}_{Yg}$.

Denote $\boldsymbol{b}_{Yg}^* = \boldsymbol{b}_{Yg} - b_0 \cdot \boldsymbol{v}$, so $\boldsymbol{b}_{Yg}^* \sim N(0, \sigma_0^2 \boldsymbol{\Sigma}^2)$. Then $\frac{\boldsymbol{\rho}_{Yg} - b_0 \cdot \boldsymbol{v}}{\boldsymbol{\rho}_{Xg}} = K_{YX} + \frac{\boldsymbol{b}_{Yg}^*}{\boldsymbol{\rho}_{Xg}}$, using Delta Method we will have:

$$\frac{\boldsymbol{r}_{Yg} - b_0 \cdot \boldsymbol{v}}{\boldsymbol{r}_{Xg}}_{|\boldsymbol{\rho}_{Yg}, \boldsymbol{\rho}_{Xg}} \sim N\left(\begin{pmatrix} \frac{\rho_{Yg_1} - b_0 \cdot v_1}{\rho_{Xg_1}} \\ \vdots \\ \frac{\rho_{Yg_m} - b_0 \cdot v_m}{\rho_{Xg_m}} \end{pmatrix}, \mathbf{J}_g^T \cdot \begin{pmatrix} \frac{\mathbf{V}_{Yg}}{n_Y} & \mathbf{0}_{m\times m} \\ \mathbf{0}_{m\times m} & \frac{\mathbf{V}_{Xg}}{n_X} \end{pmatrix} \cdot \mathbf{J}_g \right) := N(\mathbf{1} \cdot K_{YX} + \frac{\boldsymbol{b}_{Yg}^*}{\boldsymbol{\rho}_{Xg}}, \mathbf{V}_{YXg}), \quad (14)$$

Here

$$\mathbf{J}_g = \begin{pmatrix} \frac{1}{r_{Xg_1}} & \cdots & 0 & -\frac{r_{Yg_1} - b_0 \cdot v_1}{r_{Xg_1}^2} & \cdots & 0 \\ \vdots & \ddots & \vdots & \vdots & \ddots & \vdots \\ 0 & \cdots & \frac{1}{r_{Xg_m}} & 0 & \cdots & -\frac{r_{Yg_m} - b_0 \cdot v_m}{r_{Xg_m}^2} \end{pmatrix} \quad (15)$$

So we have:

$$\frac{\boldsymbol{r}_{Yg} - b_0 \cdot \boldsymbol{v}}{\boldsymbol{r}_{Xg}} = \begin{pmatrix} \frac{r_{Yg_1} - b_0 \cdot v_1}{r_{Xg_1}} \\ \vdots \\ \frac{r_{Yg_m} - b_0 \cdot v_m}{r_{Xg_m}} \end{pmatrix} \sim N(\mathbf{1} \cdot K_{YX}, \mathbf{V}_{YXg} + \sigma_0^2 \mathbf{P}) = N(\mathbf{1} \cdot K_{YX}, \mathbf{V}_{YX}) \quad (16)$$

Here

$$\mathbf{P} = \begin{pmatrix} \frac{1}{r_{Xg_1}} & & \\ & \ddots & \\ & & \frac{1}{r_{Xg_m}} \end{pmatrix} \boldsymbol{\Sigma}^2 \begin{pmatrix} \frac{1}{r_{Xg_1}} & & \\ & \ddots & \\ & & \frac{1}{r_{Xg_m}} \end{pmatrix} \quad (17)$$

Again, similar to CD-Egger, we can use iterative method to estimate (b_0, K_{YX}) and σ_0^2, and get standard errors of the estimations $se(\hat{b}_0), se(\hat{K}_{YX})$. Equation (16) is equivalent to

$$\frac{\boldsymbol{r}_{Yg}}{\boldsymbol{r}_{Xg}} = b_0 \cdot \frac{\boldsymbol{v}}{\boldsymbol{r}_{Xg}} + K_{YX} \cdot \mathbf{1} + \boldsymbol{\varepsilon}, \boldsymbol{\varepsilon} \sim N(0, \mathbf{V}_{YXg} + \sigma_0^2 \mathbf{P}) \quad (18)$$

Set initial $b_0 = 0$, $\sigma_0^2 = 0$.

Step (1): Given b_0, σ_0^2, we can get $\mathbf{V}_{YX} = \mathbf{V}_{YXg} + \sigma_0^2 \mathbf{P}$. Then estimating (b_0, K_{YX}) is a Generalize Least Square problem with design matrix $\mathbf{X}$ and covariance matrix of errors $\boldsymbol{\Omega}$:

$$\begin{aligned} \mathbf{X} &= \begin{pmatrix} \frac{\boldsymbol{v}}{\boldsymbol{r}_{Xg}} & \mathbf{1} \end{pmatrix} = \begin{pmatrix} \frac{v_1}{r_{Xg_1}} & 1 \\ \vdots & \vdots \\ \frac{v_m}{r_{Xg_m}} & 1 \end{pmatrix} \\ \boldsymbol{\Omega} &= \mathbf{V}_{YX} \end{aligned} \quad (19)$$

So we can get the estimation $(\hat{b}_0, \hat{K}_{YX})$ and their covariance matrix:

$$\begin{aligned} \begin{pmatrix} \hat{b}_0 \\ \hat{K}_{YX} \end{pmatrix} &= (\mathbf{X}^T \boldsymbol{\Omega}^{-1} \mathbf{X})^{-1} \mathbf{X}^T \boldsymbol{\Omega}^{-1} \frac{\boldsymbol{r}_{Yg}}{\boldsymbol{r}_{Xg}} \\ cov \begin{pmatrix} \hat{b}_0 \\ \hat{K}_{YX} \end{pmatrix} &= (\mathbf{X}^T \boldsymbol{\Omega}^{-1} \mathbf{X})^{-1} \end{aligned} \quad (20)$$

Step (2): Given $(\hat{b}_0, \hat{K}_{YX})$, plug them in equation (18), denoting

$$\boldsymbol{x}_{YX} = \frac{\boldsymbol{r}_{Yg}}{\boldsymbol{r}_{Xg}} - \hat{b}_0 \cdot \frac{\boldsymbol{v}}{\boldsymbol{r}_{Xg}} - \hat{K}_{YX} \cdot \mathbf{1}$$
$$\mathbf{U} = \begin{pmatrix} r_{Xg_1} & & \\ & \ddots & \\ & & r_{Xg_m} \end{pmatrix} \tag{21}$$

We get

$$\mathbf{\Sigma}^{-1}\mathbf{U}\boldsymbol{x}_{YX} \sim N(0, \mathbf{\Sigma}^{-1}\mathbf{U}\mathbf{V}_{YXg}\mathbf{U}\mathbf{\Sigma}^{-1} + \sigma_0^2\mathbf{I}) \tag{22}$$

Do the eigenvalue decomposition of

$$\mathbf{\Sigma}^{-1}\mathbf{U}\mathbf{V}_{YXg}\mathbf{U}\mathbf{\Sigma}^{-1} = \mathbf{Q}^T \begin{pmatrix} \lambda_1 & \dots & 0 \\ \vdots & \ddots & \vdots \\ 0 & \dots & \lambda_m \end{pmatrix} \mathbf{Q} \tag{23}$$

Here $\mathbf{Q}$ is orthonormal, $\lambda_1, \cdots, \lambda_m$ are eigen-values. Denote $\mathbf{Q}\mathbf{\Sigma}^{-1}\mathbf{U}\boldsymbol{x}_{YX} = \boldsymbol{x}$, we get:

$$\boldsymbol{x} \sim N(0, \begin{pmatrix} \lambda_1 + \sigma_0^2 & \dots & 0 \\ \vdots & \ddots & \vdots \\ 0 & \dots & \lambda_m + \sigma_0^2 \end{pmatrix}) \tag{24}$$

Then the log-likelihood function

$$l = -\frac{1}{2}(\frac{x_1^2}{\lambda_1 + \sigma_0^2} + \cdots + \frac{x_m^2}{\lambda_m + \sigma_0^2}) - \frac{1}{2}(\ln(\lambda_1 + \sigma_0^2) + \cdots + \ln(\lambda_m + \sigma_0^2)) + Constant \tag{25}$$

To get the MLE of σ_0^2, it is equivalent to get root of

$$\frac{x_1^2}{(\lambda_1 + \sigma_0^2)^2} + \cdots + \frac{x_m^2}{(\lambda_m + \sigma_0^2)^2} = \frac{1}{\lambda_1 + \sigma_0^2} + \cdots + \frac{1}{\lambda_1 + \sigma_0^2} \tag{26}$$

We can use the bisection method to search root for (26) and get the root, i.e., MLE of σ_0^2, denoted as $\hat{\sigma}_0^2$.

Step (3): Update $\mathbf{V}_{YX}$ with $\hat{b}_0$ and $\hat{\sigma}_0^2$, then repeat Step (1) and Step (2) iteratively until convergence, to get the final estimations $(\hat{b}_0, \hat{K}_{YX})$ and $\hat{\sigma}_0^2$. And we can get $se(\hat{b}_0)$ and $se(\hat{K}_{YX})$ from (20).
