# Supplementary material for: Inferring causal direction between two traits in the presence of horizontal pleiotropy with GWAS summary data
Source: PLoS Genet. 2020 Nov 2;16(11):e1009105. doi: 10.1371/journal.pgen.1009105 (PMC7660933; doi:10.1371/journal.pgen.1009105)
Supplement: S3 Text — (PDF) [file pgen.1009105.s003.pdf]

S3 Text: Degrees of Freedom for the Goodness-of-Fit Tests

CD-Ratio

For **CD-Ratio**, from the main text we have:

$$\frac{\boldsymbol{r}_{Yg}}{\boldsymbol{r}_{Xg}} \sim N(\mathbf{1}\cdot K_{YX}, \mathbf{V}_{YXg}) \tag{1}$$

And

$$\hat{K}_{YX} = \frac{\mathbf{1}^T \cdot \mathbf{V}_{YXg}^{-1} \cdot \frac{\mathbf{r}_{Yg}}{\mathbf{r}_{Xg}}}{\mathbf{1}^T \cdot \mathbf{V}_{YXg}^{-1} \cdot \mathbf{1}} \tag{2}$$

For simplicity, we use $\boldsymbol{r}$ to represent $\frac{\boldsymbol{r}_{Yg}}{\boldsymbol{r}_{Xg}}$, K to represent K_{YX}, $\hat{K}$ to represent $\hat{K}_{YX}$, and $\mathbf{V}$ to represent $\mathbf{V}_{YXg}$. Then the test statistic Q_{Ratio} is:

$$Q_{Ratio} = \left(\boldsymbol{r} - \mathbf{1}\cdot\hat{K}\right)^T \mathbf{V}^{-1} \left(\boldsymbol{r} - \mathbf{1}\cdot\hat{K}\right) \tag{3}$$

And we have

$$\boldsymbol{r} - \mathbf{1}\cdot\hat{K} = \left(\mathbf{I} - \frac{\mathbf{1}\mathbf{1}^T\mathbf{V}^{-1}}{\mathbf{1}^T\mathbf{V}^{-1}\mathbf{1}}\right)\boldsymbol{r} \tag{4}$$

So

$$\mathbf{V}^{-\frac{1}{2}}(\boldsymbol{r} - \mathbf{1}\cdot\hat{K}) = \mathbf{V}^{-\frac{1}{2}}\left(\mathbf{I} - \frac{\mathbf{1}\mathbf{1}^T\mathbf{V}^{-1}}{\mathbf{1}^T\mathbf{V}^{-1}\mathbf{1}}\right)\boldsymbol{r} \sim N(\boldsymbol{\mu}, \mathbf{A}) \tag{5}$$

Here

$$\boldsymbol{\mu} = \mathbf{V}^{-\frac{1}{2}}\left(\mathbf{I} - \frac{\mathbf{1}\mathbf{1}^T\mathbf{V}^{-1}}{\mathbf{1}^T\mathbf{V}^{-1}\mathbf{1}}\right)\mathbf{1}\cdot K = \mathbf{0} \tag{6}$$

And

$$\begin{aligned}\mathbf{A} &= \mathbf{V}^{-\frac{1}{2}}\left(\mathbf{I} - \frac{\mathbf{1}\mathbf{1}^T\mathbf{V}^{-1}}{\mathbf{1}^T\mathbf{V}^{-1}\mathbf{1}}\right)\mathbf{V}\left(\mathbf{I} - \frac{\mathbf{V}^{-1}\mathbf{1}\mathbf{1}^T}{\mathbf{1}^T\mathbf{V}^{-1}\mathbf{1}}\right)\mathbf{V}^{-\frac{1}{2}} \\ &= \mathbf{I} - \frac{\mathbf{V}^{-\frac{1}{2}}\mathbf{1}\mathbf{1}^T\mathbf{V}^{-\frac{1}{2}}}{\mathbf{1}^T\mathbf{V}^{-1}\mathbf{1}}\end{aligned} \tag{7}$$

Since $\frac{\mathbf{V}^{-\frac{1}{2}}\mathbf{1}\mathbf{1}^T\mathbf{V}^{-\frac{1}{2}}}{\mathbf{1}^T\mathbf{V}^{-1}\mathbf{1}}$ is the projection matrix to vector $\mathbf{V}^{-\frac{1}{2}}\mathbf{1}$, eigenvalues of $\mathbf{A}$ are $(m-1)$ 1's and one 0, leading to $Q_{Ratio} \sim \chi^2_{m-1}$.

CD-Egger

For **CD-Egger**, from the main text we have:

$$\boldsymbol{r}_{Yg} = b_0\cdot\boldsymbol{v} + K_{YX}\cdot\boldsymbol{r}_{Xg} + \boldsymbol{\varepsilon}, \boldsymbol{\varepsilon} \sim N(0, \frac{\mathbf{V}_{Yg}}{n_Y} + \sigma_0^2\boldsymbol{\Sigma}^2 = \mathbf{V}_{YX}) \tag{8}$$

As we show in the Section 3 above, plug in estimation $\hat{\sigma}_0^2$ of σ_0^2 we get $\mathbf{V}_{YX}$, with notations in equation (6) of S2 Text, we have

$$\begin{pmatrix}\hat{b}_0 \\ \hat{K}_{YX}\end{pmatrix} = (\mathbf{X}^T\boldsymbol{\Omega}^{-1}\mathbf{X})^{-1}\mathbf{X}^T\boldsymbol{\Omega}^{-1}\boldsymbol{r}_{Yg} \tag{9}$$

And the test statistic Q_{Egger} is:

$$Q_{Egger} = (\boldsymbol{r}_{Yg} - \hat{b}_0\cdot\boldsymbol{v} - \hat{K}_{YX}\cdot\boldsymbol{r}_{Xg})^T\mathbf{V}_{YX}^{-1}(\boldsymbol{r}_{Yg} - \hat{b}_0\cdot\boldsymbol{v} - \hat{K}_{YX}\cdot\boldsymbol{r}_{Xg}) \tag{10}$$

Here

$$\begin{aligned} & \boldsymbol{r}_{Yg} - \hat{b}_0 \cdot \boldsymbol{v} - \hat{K}_{YX} \cdot \boldsymbol{r}_{Xg} \\ = & \left(\mathbf{I} - \mathbf{X}(\mathbf{X}^T \boldsymbol{\Omega}^{-1} \mathbf{X})^{-1} \mathbf{X}^T \boldsymbol{\Omega}^{-1}\right) \boldsymbol{r}_{Yg} \\ = & \left(\mathbf{I} - \mathbf{X}(\mathbf{X}^T \boldsymbol{\Omega}^{-1} \mathbf{X})^{-1} \mathbf{X}^T \boldsymbol{\Omega}^{-1}\right) \boldsymbol{\varepsilon} \end{aligned} \tag{11}$$

So

$$\begin{aligned} & \mathbf{V}_{YX}^{-\frac{1}{2}} (\boldsymbol{r}_{Yg} - \hat{b}_0 \cdot \boldsymbol{v} - \hat{K}_{YX} \cdot \boldsymbol{r}_{Xg}) \\ = & \mathbf{V}_{YX}^{-\frac{1}{2}} \left(\mathbf{I} - \mathbf{X}(\mathbf{X}^T \boldsymbol{\Omega}^{-1} \mathbf{X})^{-1} \mathbf{X}^T \boldsymbol{\Omega}^{-1}\right) \boldsymbol{\varepsilon} \sim N(\mathbf{0}, \mathbf{A}) \end{aligned} \tag{12}$$

Here

$$\begin{aligned} \mathbf{A} &= \mathbf{V}_{YX}^{-\frac{1}{2}} \left(\mathbf{I} - \mathbf{X}(\mathbf{X}^T \boldsymbol{\Omega}^{-1} \mathbf{X})^{-1} \mathbf{X}^T \boldsymbol{\Omega}^{-1}\right) \mathbf{V}_{YX} \left(\mathbf{I} - \boldsymbol{\Omega}^{-1} \mathbf{X}(\mathbf{X}^T \boldsymbol{\Omega}^{-1} \mathbf{X})^{-1} \mathbf{X}^T\right) \mathbf{V}_{YX}^{-\frac{1}{2}} \\ &= \mathbf{I} - \boldsymbol{\Omega}^{-\frac{1}{2}} \mathbf{X}(\mathbf{X}^T \boldsymbol{\Omega}^{-1} \mathbf{X})^{-1} \mathbf{X}^T \boldsymbol{\Omega}^{-\frac{1}{2}} \end{aligned} \tag{13}$$

Since $\boldsymbol{\Omega}^{-\frac{1}{2}} \mathbf{X}(\mathbf{X}^T \boldsymbol{\Omega}^{-1} \mathbf{X})^{-1} \mathbf{X}^T \boldsymbol{\Omega}^{-\frac{1}{2}}$ is the projection matrix to dimension-2 space $\boldsymbol{\Omega}^{-\frac{1}{2}} \mathbf{X}$, eigenvalues of $\mathbf{A}$ are $(m-2)$ 2's and two 0's, leading to $Q_{Egger} \sim \chi^2_{m-2}$.

CD-GLS

Based on the asymptotic distribution from above equation (16) in S2 Text, for CD-GLS we can construct GOF test:

$$Q_{GLS} = \left(\frac{\boldsymbol{r}_{Yg} - \hat{b}_0 \cdot \boldsymbol{v}}{\boldsymbol{r}_{Xg}} - \mathbf{1} \cdot \hat{K}_{YX}\right)^T \mathbf{V}_{YX}^{-1} \left(\frac{\boldsymbol{r}_{Yg} - \hat{b}_0 \cdot \boldsymbol{v}}{\boldsymbol{r}_{Xg}} - \mathbf{1} \cdot \hat{K}_{YX}\right) \tag{14}$$

Similar to CD-Egger, the (asymptotic) null distribution is $Q_{GLS} \sim \chi^2_{m-2}$.
