# Supplementary material for: Inferring causal direction between two traits in the presence of horizontal pleiotropy with GWAS summary data
Source: PLoS Genet. 2020 Nov 2;16(11):e1009105. doi: 10.1371/journal.pgen.1009105 (PMC7660933; doi:10.1371/journal.pgen.1009105)
Supplement: S4 Text — (PDF) [file pgen.1009105.s004.pdf]

S4 Text: More Results for LDL and CAD

Removing Locus 253 and Locus 1246

For the real data example of LDL and CAD, we showed the results using 22 SNPs from 12 loci in the main text, Locus 253 and Locus 1246 showed negative effects of LDL on CAD. We can remove these 2 loci, then apply CD-Ratio, CD-Egger and CD-GLS to the rest 20 SNPs from the 10 loci. Figure A shows the corresponding results as forest plots, and Table A shows 95% confidence intervals for the combined results.

Figure A: Forest plots for inferring the causal direction between LDL and CAD, showing SNP and locus-specific results and the combined results across 20 SNPs from 10 loci, after removing Locus 253 and Locus 1246.

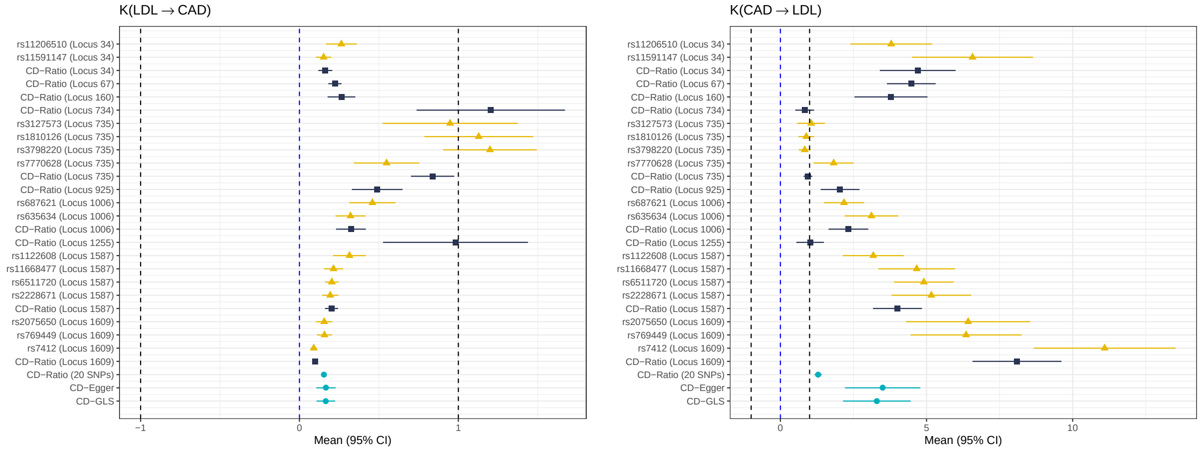

Table A: Results of inferring the causal direction between LDL and CAD after combining across 20 SNPs in 10 loci after removing Locus 253 and Locus 1246.

	LDL → *CAD*		*CAD* → *LDL*	
Method	$\hat{K}$ [95% CI]	$\hat{b}_0$ (SE)	$\hat{K}$ [95% CI]	$\hat{b}_0$ (SE)
CD-Ratio	0.154 [0.140, 0.168]	NA	1.293 [1.168, 1.417]	NA
CD-Egger	0.167 [0.106, 0.228]	0.005 (0.002)	3.500 [2.209, 4.790]	-0.021 (0.010)
CD-GLS	0.166 [0.107, 0.225]	0.005 (0.002)	3.301 [2.144, 4.457]	-0.019 (0.009)

Only Using Independent SNPs

For the real data example of LDL and CAD, we showed the results using 22 SNPs from 12 loci in the main text. We can only pick the most significant SNP at each of 12 loci according to the combined significance level with LDL and CAD, as described in Section 2.7 in the main text, then we get 12 independent SNPs from the 12 loci. We can apply CD-Ratio to each SNPs and all 12 SNPs, and apply CD-Egger and CD-GLS to all 12 SNPs. Figure B shows the corresponding results as forest plots, and Table B shows 95% confidence intervals for the combined results.

Figure B: Forest plots for inferring the causal direction between LDL and CAD, showing SNP results and the combined results across 12 independent SNPs from 12 loci

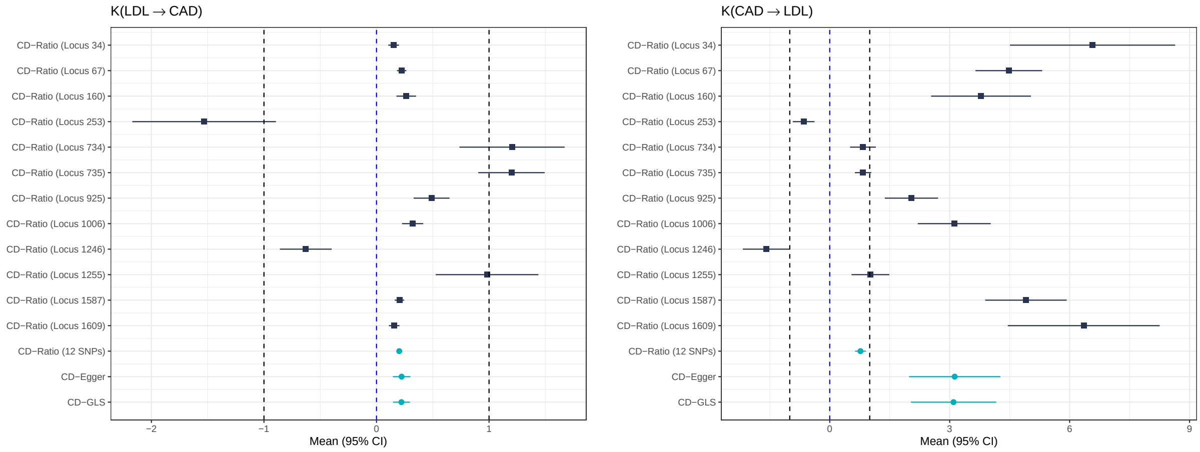

Table B: Results of inferring the causal direction between LDL and CAD after combining across 12 independent SNPs in 12 loci.

	LDL → *CAD*		*CAD* → *LDL*	
Method	$\hat{K}$ [95% CI]	$\hat{b}_0$ (SE)	$\hat{K}$ [95% CI]	$\hat{b}_0$ (SE)
CD-Ratio	0.203 [0.183, 0.223]	NA	0.769 [0.633, 0.904]	NA
CD-Egger	0.223 [0.145, 0.301]	0.007 (0.002)	3.126 [1.983, 4.268]	-0.023 (0.010)
CD-GLS	0.222 [0.147, 0.297]	0.007 (0.002)	3.097 [2.029, 4.165]	-0.025 (0.009)
