# Supplementary material for: Inferring causal direction between two traits in the presence of horizontal pleiotropy with GWAS summary data
Source: PLoS Genet. 2020 Nov 2;16(11):e1009105. doi: 10.1371/journal.pgen.1009105 (PMC7660933; doi:10.1371/journal.pgen.1009105)
Supplement: S5 Text — (PDF) [file pgen.1009105.s005.pdf]

S5 Text: Results for LDL/HDL and CAD with Multivariable Approaches

LDL and CAD: MV-CD-Egger (adjusting for HDL)

For LDL and CAD, we started with the 22 SNPs from 12 independent loci as described in the main text. Among the p-values of these 22 SNPs for HDL, 8 of them were smaller than 5×10^{-8}, 2 of them between 5×10^{-8} and 5×10^{-6}, 3 of them between 5×10^{-6} and 5×10^{-4}, and 9 of them larger than 5×10^{-4}. We could use either all these 22 SNPs or only the 8 SNPs significant for all 3 traits. Table A and Table B show the results (after adjusting for HDL) with the two sets of the SNPs as IVs respectively.

Table A: The results for inferring the causal direction between LDL and CAD combining across all 22 SNPs in 12 loci, and adjusting for HDL.

	$LDL \to CAD$		$CAD \to LDL$	
Method	$\hat{K}$ [95% CI]	$\hat{b}_0$ (SE)	$\hat{K}$ [95% CI]	$\hat{b}_0$ (SE)
CD-Egger	0.172 $[0.078, 0.267]$	0.006 (0.002)	2.158 $[0.996, 3.319]$	-0.020 (0.008)

Table B: The results for inferring the causal direction between LDL and CAD combining across 8 SNPs from 6 loci, and adjusting for HDL.

	$LDL \to CAD$		$CAD \to LDL$	
Method	$\hat{K}$ [95% CI]	$\hat{b}_0$ (SE)	$\hat{K}$ [95% CI]	$\hat{b}_0$ (SE)
CD-Egger	0.114 $[-0.008, 0.235]$	0.003 (0.004)	2.680 $[-0.099, 5.458]$	-0.033 (0.014)

We can see that, using the 22 SNPs, the 95% CI of K for $LDL \to CAD$ was [0.078,0.267], completely inside (0,1], while the 95% for $CAD \to LDL$ was [0.996,3.319], almost completely outside [-1,1]. So we have strong evidence to say that, after adjusting for HDL, we conclude that the causal direction is from LDL to CAD. Using the 8 SNPs, although the lengths of the confidence intervals were larger so we could not make a conclusion, the point estimate of K for $LDL \to CAD$ was 0.114, inside (0,1] while that for $CAD \to LDL$ was 2.680, larger than 1 giving some evidence to support the causal effect of LDL on CAD.

HDL and CAD: MV-CD-Egger (after adjusting for LDL)

For HDL and CAD, we selected the 8 SNPs from 6 independent loci as described in the main text, and all these 8 SNPs had p-values less than 5×10^{-8} for LDL. Table C shows the results (after adjusting for LDL).

Table C: The results for inferring the causal direction between HDL and CAD combining across all 8 SNPs in 6 loci, and adjusting for LDL.

	$HDL \to CAD$		$CAD \to HDL$	
Method	$\hat{K}$ [95% CI]	$\hat{b}_0$ (SE)	$\hat{K}$ [95% CI]	$\hat{b}_0$ (SE)
CD-Egger	-0.181 $[-0.531, 0.168]$	0.003 (0.004)	-0.660 $[-1.903, 0.584]$	-0.007 (0.007)

We can see that, for both directions the 95% CIs covered 0, and both point estimates were in [-1,0), so we cannot conclude with any causal relationship between HDL and CAD.

MV-MR Results

Using R package **TwoSampleMR**, we applied two multivariable MR methods: the first one was MV-IVW with function `mv_ivw()` and the second MV-MR-Egger with `mv_multiple()` with an intercept term. Table D shows the results for CAD, LDL and HDL with each as the outcome respectively. As before, we used 2017 lipid data and 2017 CAD data. We set the significance cutoff at 5×10^{-8} to choose independent SNPs as instruments. When CAD was the outcome we used 169 IVs: 89 significant for LDL, 106 significant for HDL, and 26 for both; when LDL was the outcome, we use 142 IVs: 34 significant for CAD, 114 significant for HDL, and 6 for both; when HDL was the outcome, we used 127 IVs: 38 significant for CAD, 101 significant for LDL, and 12 for both.

Table D: Multivariable MR results with one of CAD, LDL and HDL as the outcome and the other two as exposure based on 2017 lipids GWAS summary data and 2017 CAD GWAS summary data.

CAD as outcome						
	MV-MR-IVW			MV-MR-Egger		
Covariate	$\hat{\beta}$	SE($\hat{\beta}$)	p-value	$\hat{\beta}$	SE($\hat{\beta}$)	p-value
LDL	0.492	0.053	1.1×10^{-20}	0.485	0.047	7.2×10^{-25}
HDL	-0.219	0.045	1.2×10^{-6}	-0.214	0.044	1.2×1.2^{-6}
**LDL as outcome**						
	MV-MR-IVW			MV-MR-Egger		
Covariate	$\hat{\beta}$	SE($\hat{\beta}$)	p-value	$\hat{\beta}$	SE($\hat{\beta}$)	p-value
CAD	0.187	0.148	0.206	0.402	0.077	1.7×10^{-7}
HDL	0.134	0.077	0.083	0.034	0.080	0.673
**HDL as outcome**						
	MV-MR-IVW			MV-MR-Egger		
Covariate	$\hat{\beta}$	SE($\hat{\beta}$)	p-value	$\hat{\beta}$	SE($\hat{\beta}$)	p-value
CAD	-0.044	0.033	0.177	-0.125	0.074	0.092
LDL	0.029	0.085	0.736	-0.034	0.066	0.614

Through R package **TwoSampleMR**, the functions can automatically extracts IVs from 2013 lipid data and 2015 CAD data from its database, we show the corresponding results in Table E. We set cutoff 5×10^{-8} to choose independent SNPs as instruments, when CAD is outcome we use 132 IVs, 68 significant for LDL, 75 significant for HDL, and 11 for both; when LDL is outcome we use 102 IVs, 26 significant for CAD, 79 significant for HDL, and 3 for both; when HDL is outcome we use 95 IVs, 26 significant for CAD, 74 significant for LDL, and 5 for both.

Table E: Multivariable MR results with one of CAD, LDL, HDL as outcome and the other two as covariates, using 2013 lipids GWAS summary data and 2015 CAD GWAS summary data.

CAD as outcome						
	MV-MR-IVW			MV-MR-Egger		
Covariate	$\hat{\beta}$	SE($\hat{\beta}$)	p-value	$\hat{\beta}$	SE($\hat{\beta}$)	p-value
LDL	0.397	0.056	1.3×10^{-12}	0.389	0.050	8.4×10^{-15}
HDL	-0.142	0.054	8.1×10^{-3}	-0.141	0.057	0.014
**LDL as outcome**						
	MV-MR-IVW			MV-MR-Egger		
Covariate	$\hat{\beta}$	SE($\hat{\beta}$)	p-value	$\hat{\beta}$	SE($\hat{\beta}$)	p-value
CAD	0.067	0.070	0.336	0.144	0.057	0.011
HDL	0.017	0.066	0.797	-0.045	0.063	0.479
**HDL as outcome**						
	MV-MR-IVW			MV-MR-Egger		
Covariate	$\hat{\beta}$	SE($\hat{\beta}$)	p-value	$\hat{\beta}$	SE($\hat{\beta}$)	p-value
CAD	-0.018	0.028	0.513	-0.054	0.073	0.459
LDL	-0.039	0.102	0.700	-0.080	0.071	0.258

Results in Table D and Table E are similar. We can see at significant level 0.05, both MV-MR methods would conclude with LDL and HDL has causal effect on CAD, and MV-MR-Egger concludes CAD has causal effect on LDL.
