# Supplementary material for: Inferring causal direction between two traits in the presence of horizontal pleiotropy with GWAS summary data
Source: PLoS Genet. 2020 Nov 2;16(11):e1009105. doi: 10.1371/journal.pgen.1009105 (PMC7660933; doi:10.1371/journal.pgen.1009105)

S6 Text: More Simulation Results

Details of Simulation Setups

We used three parameters $(\beta_{YX}, \mu_\alpha, \sigma_\alpha)$ in each simulation setup. β_{YX} controls the causal effect size, μ_α controls the mean direct effect size, σ_α controls the variation of the (random) direct effect sizes. We tried 75 different combinations of $\beta_{YX} = -0.2, -0.1, 0, 0.1, 0.2, \mu_\alpha = 0, 0.2, 0.4, 0.6, 1, \sigma_\alpha = 0, 0.1, 0.2$. For each setup we first generated $\boldsymbol{\beta}_{Xg} = (\beta_{Xg_1}, \beta_{Xg_2}, ..., \beta_{Xg_{22}})^T$, the vector of the effect sizes of the 22 SNPs on X, with each element independently drawn from a truncated standard normal distribution (truncated at 0.5 to ensure $|\beta_{Xg_j}| \geq 0.5$). Then in each simulation, we generated the two traits from the true models:

$$X_i = \boldsymbol{Z}_i \boldsymbol{\beta}_{Xg} + U_i + \varepsilon_{Xi}, \qquad Y_i = \beta_{YX} \cdot X_i + \boldsymbol{Z}_i \boldsymbol{\alpha} + U_i + \varepsilon_{Yi},$$

where $\boldsymbol{Z}_i$ was the (row) vector of the genotype scores of the 22 SNPs for subject $i = 1, 2, ..., n$, $U_i \sim N(0,1)$ was a confounder; the direct effects of the SNPs on Y, $\boldsymbol{\alpha} = (\alpha_1, ..., \alpha_{22})^T$, were generated independently $\alpha_i \sim N(\mu_\alpha, \sigma_\alpha^2)$; and both error terms $\varepsilon_{Xi}, \varepsilon_{Yi}$ were independently from $N(0,4)$.

Estimating K_{YX}

Table A shows simulation results for estimating K_{YX} of CD-Ratio, CD-Egger, and CD-GLS, for the setup $\mu_\alpha = 0$, $\sigma_\alpha = 0$, and various β_{YX} levels. In each row we list the value β_{YX} in the first column, and corresponding true value K_{YX} in the second column, and for each of three methods we show the estimation results similar to Table 5 in the main text. Table B to Table O show similar results for other setups. When $\beta_{YX} \neq 0$ the true causal direction is from X to Y, we can calculate the true value of $K_{YX} = \beta_{YX} \cdot \frac{\sqrt{var(X)}}{\sqrt{var(Y)}}$, here

$$\begin{aligned} var(X) &= \boldsymbol{\beta}_{Xg}^T \boldsymbol{\Sigma} \boldsymbol{\beta}_{Xg} + var(U) + var(\varepsilon_X) \\ var(Y) &= (\mu_\alpha \cdot \mathbf{1} + \beta_{YX} \cdot \boldsymbol{\beta}_{Xg})^T \boldsymbol{\Sigma} (\mu_\alpha \cdot \mathbf{1} + \beta_{YX} \cdot \boldsymbol{\beta}_{Xg}) + (\beta_{YX}+1)^2 var(U) + \beta_{YX}^2 var(\varepsilon_X) + var(\varepsilon_Y) \end{aligned} \quad (1)$$

$\boldsymbol{\Sigma}$ is the covariance matrix of SNPs. When $\beta_{YX} = 0$, we have $K_{YX} = 0$.

Table A: Results of $\mu_\alpha = 0$, $\sigma_\alpha = 0$: K_{YX}

		CD-Ratio			CD-Egger			CD-GLS		
β_{YX}	K_{YX}	Mean$(\hat{K})$	sd$(\hat{K})$	Mean$(se(\hat{K}))$	Mean$(\hat{K})$	sd$(\hat{K})$	Mean$(se(\hat{K}))$	Mean$(\hat{K})$	sd$(\hat{K})$	Mean$(se(\hat{K}))$
-0.2	-0.442	-0.4415	0.0028	0.0036	-0.4415	0.0028	0.0035	-0.4415	0.0028	0.0036
-0.1	-0.2344	-0.2341	0.0032	0.0036	-0.2341	0.0032	0.0036	-0.2341	0.0032	0.0036
0	0	0.0001	0.0034	0.0035	0.0001	0.0034	0.0036	0.0001	0.0034	0.0036
0.1	0.2257	0.2256	0.0033	0.0036	0.2256	0.0033	0.0036	0.2256	0.0033	0.0036
0.2	0.4139	0.4137	0.003	0.0036	0.4137	0.003	0.0035	0.4137	0.003	0.0036

Table B: Results of $\mu_\alpha = 0.2$, $\sigma_\alpha = 0$: K_{YX}

		CD-Ratio			CD-Egger			CD-GLS		
β_{YX}	K_{YX}	Mean$(\hat{K})$	sd$(\hat{K})$	Mean$(se(\hat{K}))$	Mean$(\hat{K})$	sd$(\hat{K})$	Mean$(se(\hat{K}))$	Mean$(\hat{K})$	sd$(\hat{K})$	Mean$(se(\hat{K}))$
-0.2	-0.3995	-0.4106	0.003	0.0038	-0.3992	0.0026	0.0034	-0.3992	0.0026	0.0035
-0.1	-0.2093	-0.2191	0.0032	0.0039	-0.2091	0.0029	0.0035	-0.2091	0.0029	0.0035
0	0	-0.0097	0.0034	0.0039	0	0.0031	0.0035	0	0.0031	0.0035
0.1	0.2027	0.1947	0.0034	0.0039	0.2026	0.003	0.0035	0.2026	0.003	0.0036
0.2	0.3773	0.3675	0.0031	0.0039	0.3771	0.0028	0.0035	0.3771	0.0028	0.0036

Table C: Results of $\mu_\alpha = 0.4$, $\sigma_\alpha = 0$: K_{YX}

		CD-Ratio			CD-Egger			CD-GLS		
β_{YX}	K_{YX}	Mean($\hat{K}$)	sd($\hat{K}$)	Mean$(se(\hat{K}))$	Mean($\hat{K}$)	sd($\hat{K}$)	Mean$(se(\hat{K}))$	Mean($\hat{K}$)	sd($\hat{K}$)	Mean$(se(\hat{K}))$
-0.2	-0.3203	-0.3298	0.0031	0.004	-0.3202	0.0022	0.0032	-0.3202	0.0022	0.0033
-0.1	-0.1649	-0.1777	0.0032	0.0041	-0.1648	0.0023	0.0033	-0.1648	0.0023	0.0033
0	0	-0.0158	0.0033	0.0041	0	0.0025	0.0033	0	0.0025	0.0034
0.1	0.1614	0.1464	0.0033	0.0041	0.1613	0.0025	0.0034	0.1613	0.0025	0.0034
0.2	0.3079	0.2956	0.0032	0.0041	0.3078	0.0024	0.0034	0.3078	0.0024	0.0035

Table D: Results of $\mu_\alpha = 0.6$, $\sigma_\alpha = 0$: K_{YX}

		CD-Ratio			CD-Egger			CD-GLS		
β_{YX}	K_{YX}	Mean($\hat{K}$)	sd($\hat{K}$)	Mean$(se(\hat{K}))$	Mean($\hat{K}$)	sd($\hat{K}$)	Mean$(se(\hat{K}))$	Mean($\hat{K}$)	sd($\hat{K}$)	Mean$(se(\hat{K}))$
-0.2	-0.2535	-0.2713	0.0033	0.0041	-0.2535	0.0018	0.0031	-0.2535	0.0018	0.0032
-0.1	-0.129	-0.1538	0.0034	0.0042	-0.129	0.0019	0.0031	-0.129	0.0019	0.0032
0	0	-0.0304	0.0034	0.0042	0	0.002	0.0032	0	0.002	0.0032
0.1	0.1272	0.0955	0.0034	0.0042	0.1272	0.002	0.0033	0.1272	0.002	0.0033
0.2	0.2469	0.2181	0.0033	0.0042	0.2468	0.002	0.0034	0.2468	0.002	0.0034

Table E: Results of $\mu_\alpha = 1$, $\sigma_\alpha = 0$: K_{YX}

		CD-Ratio			CD-Egger			CD-GLS		
β_{YX}	K_{YX}	Mean($\hat{K}$)	sd($\hat{K}$)	Mean$(se(\hat{K}))$	Mean($\hat{K}$)	sd($\hat{K}$)	Mean$(se(\hat{K}))$	Mean($\hat{K}$)	sd($\hat{K}$)	Mean$(se(\hat{K}))$
-0.2	-0.171	-0.2063	0.0036	0.0042	-0.171	0.0013	0.003	-0.171	0.0012	0.003
-0.1	-0.0862	-0.1297	0.0036	0.0043	-0.0862	0.0013	0.003	-0.0862	0.0013	0.003
0	0	-0.0503	0.0036	0.0043	0	0.0013	0.0031	0	0.0013	0.0031
0.1	0.0856	0.0315	0.0035	0.0043	0.0855	0.0014	0.0032	0.0856	0.0014	0.0032
0.2	0.1687	0.1144	0.0035	0.0043	0.1687	0.0014	0.0033	0.1687	0.0014	0.0033

Table F: Results of $\mu_\alpha = 0$, $\sigma_\alpha = 0.1$: K_{YX}

		CD-Ratio			CD-Egger			CD-GLS		
β_{YX}	K_{YX}	Mean($\hat{K}$)	sd($\hat{K}$)	Mean$(se(\hat{K}))$	Mean($\hat{K}$)	sd($\hat{K}$)	Mean$(se(\hat{K}))$	Mean($\hat{K}$)	sd($\hat{K}$)	Mean$(se(\hat{K}))$
-0.2	-0.442	-0.4254	0.0477	0.0037	-0.4335	0.0345	0.0386	-0.4335	0.0347	0.0394
-0.1	-0.2344	-0.2233	0.0545	0.0037	-0.2296	0.0409	0.0408	-0.2295	0.0414	0.0416
0	0	0.0013	0.0563	0.0037	-0.0007	0.0431	0.041	-0.0005	0.0436	0.0419
0.1	0.2257	0.2181	0.0531	0.0037	0.2201	0.0397	0.0393	0.2203	0.0402	0.0401
0.2	0.4139	0.4019	0.0459	0.0037	0.4056	0.0332	0.0362	0.4059	0.0334	0.0369

Table G: Results of $\mu_\alpha = 0.2$, $\sigma_\alpha = 0.1$: K_{YX}

		CD-Ratio			CD-Egger			CD-GLS		
β_{YX}	K_{YX}	Mean($\hat{K}$)	sd($\hat{K}$)	Mean$(se(\hat{K}))$	Mean($\hat{K}$)	sd($\hat{K}$)	Mean$(se(\hat{K}))$	Mean($\hat{K}$)	sd($\hat{K}$)	Mean$(se(\hat{K}))$
-0.2	-0.3995	-0.4048	0.0463	0.0038	-0.3936	0.0328	0.035	-0.3936	0.0326	0.0357
-0.1	-0.2093	-0.2172	0.0517	0.0039	-0.2061	0.037	0.0366	-0.206	0.0373	0.0373
0	0	-0.0112	0.0538	0.0039	-0.0005	0.0386	0.0368	-0.0004	0.0392	0.0375
0.1	0.2027	0.1898	0.0514	0.0039	0.1987	0.0369	0.0355	0.1989	0.0375	0.0362
0.2	0.3773	0.3612	0.0452	0.0039	0.3711	0.0326	0.0331	0.3714	0.0331	0.0338

Table H: Results of $\mu_\alpha = 0.4$, $\sigma_\alpha = 0.1$: K_{YX}

		CD-Ratio			CD-Egger			CD-GLS		
β_{YX}	K_{YX}	Mean($\hat{K}$)	sd($\hat{K}$)	Mean($se(\hat{K})$)	Mean($\hat{K}$)	sd($\hat{K}$)	Mean($se(\hat{K})$)	Mean($\hat{K}$)	sd($\hat{K}$)	Mean($se(\hat{K})$)
-0.2	-0.3203	-0.3301	0.0412	0.004	-0.3176	0.028	0.0282	-0.3176	0.028	0.0288
-0.1	-0.1649	-0.1796	0.0435	0.0041	-0.1636	0.0298	0.029	-0.1635	0.03	0.0296
0	0	-0.0183	0.0448	0.0041	-0.0004	0.0306	0.0291	-0.0003	0.0311	0.0297
0.1	0.1614	0.1438	0.0448	0.0041	0.1593	0.0301	0.0285	0.1595	0.0307	0.029
0.2	0.3079	0.2925	0.0424	0.0041	0.3046	0.0284	0.0272	0.3049	0.029	0.0278

Table I: Results of $\mu_\alpha = 0.6$, $\sigma_\alpha = 0.1$: K_{YX}

		CD-Ratio			CD-Egger			CD-GLS		
β_{YX}	K_{YX}	Mean($\hat{K}$)	sd($\hat{K}$)	Mean($se(\hat{K})$)	Mean($\hat{K}$)	sd($\hat{K}$)	Mean($se(\hat{K})$)	Mean($\hat{K}$)	sd($\hat{K}$)	Mean($se(\hat{K})$)
-0.2	-0.2535	-0.2721	0.0341	0.0041	-0.2524	0.0228	0.0224	-0.2524	0.0228	0.0229
-0.1	-0.129	-0.1552	0.0351	0.0042	-0.1285	0.0236	0.0228	-0.1285	0.0238	0.0233
0	0	-0.0317	0.0357	0.0042	-0.0003	0.024	0.0228	-0.0002	0.0244	0.0233
0.1	0.1272	0.0944	0.0362	0.0042	0.1261	0.024	0.0225	0.1263	0.0244	0.023
0.2	0.2469	0.217	0.0359	0.0042	0.2451	0.0234	0.0219	0.2454	0.0239	0.0224

Table J: Results of $\mu_\alpha = 1$, $\sigma_\alpha = 0.1$: K_{YX}

		CD-Ratio			CD-Egger			CD-GLS		
β_{YX}	K_{YX}	Mean($\hat{K}$)	sd($\hat{K}$)	Mean($se(\hat{K})$)	Mean($\hat{K}$)	sd($\hat{K}$)	Mean($se(\hat{K})$)	Mean($\hat{K}$)	sd($\hat{K}$)	Mean($se(\hat{K})$)
-0.2	-0.171	-0.2066	0.0236	0.0042	-0.1708	0.0157	0.0151	-0.1708	0.0158	0.0154
-0.1	-0.0862	-0.1301	0.0238	0.0043	-0.0862	0.0159	0.0153	-0.0862	0.0161	0.0156
0	0	-0.0506	0.0241	0.0043	-0.0002	0.0161	0.0153	-0.0002	0.0164	0.0156
0.1	0.0856	0.0313	0.0244	0.0043	0.0851	0.0162	0.0152	0.0852	0.0165	0.0155
0.2	0.1687	0.1143	0.0247	0.0043	0.1681	0.0161	0.015	0.1682	0.0165	0.0153

Table K: Results of $\mu_\alpha = 0$, $\sigma_\alpha = 0.2$: K_{YX}

		CD-Ratio			CD-Egger			CD-GLS		
β_{YX}	K_{YX}	Mean($\hat{K}$)	sd($\hat{K}$)	Mean($se(\hat{K})$)	Mean($\hat{K}$)	sd($\hat{K}$)	Mean($se(\hat{K})$)	Mean($\hat{K}$)	sd($\hat{K}$)	Mean($se(\hat{K})$)
-0.2	-0.442	-0.3933	0.0936	0.0038	-0.4113	0.0669	0.0727	-0.4111	0.0674	0.0742
-0.1	-0.2344	-0.2037	0.1043	0.0039	-0.2168	0.0771	0.0763	-0.2166	0.0781	0.078
0	0	0.0031	0.107	0.0038	-0.0017	0.0804	0.0768	-0.0015	0.0815	0.0784
0.1	0.2257	0.2031	0.1021	0.0038	0.2066	0.0751	0.0738	0.2068	0.076	0.0754
0.2	0.4139	0.3767	0.0909	0.0038	0.385	0.0644	0.0685	0.3853	0.0648	0.07

Table L: Results of $\mu_\alpha = 0.2$, $\sigma_\alpha = 0.2$: K_{YX}

		CD-Ratio			CD-Egger			CD-GLS		
β_{YX}	K_{YX}	Mean($\hat{K}$)	sd($\hat{K}$)	Mean($se(\hat{K})$)	Mean($\hat{K}$)	sd($\hat{K}$)	Mean($se(\hat{K})$)	Mean($\hat{K}$)	sd($\hat{K}$)	Mean($se(\hat{K})$)
-0.2	-0.3995	-0.3871	0.089	0.0039	-0.3775	0.0632	0.0668	-0.3773	0.0633	0.0682
-0.1	-0.2093	-0.2079	0.099	0.0039	-0.1971	0.0705	0.0695	-0.1968	0.0712	0.071
0	0	-0.0129	0.1023	0.0039	-0.0012	0.0733	0.0698	-0.0009	0.0744	0.0712
0.1	0.2027	0.1764	0.0977	0.0039	0.1889	0.0703	0.0675	0.1893	0.0715	0.0689
0.2	0.3773	0.3409	0.0874	0.0039	0.3555	0.0629	0.0633	0.3559	0.0639	0.0646

Table M: Results of $\mu_\alpha = 0.4$, $\sigma_\alpha = 0.2$: K_{YX}

		CD-Ratio			CD-Egger			CD-GLS		
β_{YX}	K_{YX}	Mean($\hat{K}$)	sd($\hat{K}$)	Mean$(se(\hat{K}))$	Mean($\hat{K}$)	sd($\hat{K}$)	Mean$(se(\hat{K}))$	Mean($\hat{K}$)	sd($\hat{K}$)	Mean$(se(\hat{K}))$
-0.2	-0.3203	-0.3305	0.0782	0.004	-0.3097	0.0546	0.0548	-0.3096	0.0546	0.0559
-0.1	-0.1649	-0.1836	0.0837	0.004	-0.1594	0.0578	0.0562	-0.1592	0.0584	0.0574
0	0	-0.0248	0.0871	0.004	-0.0009	0.0593	0.0563	-0.0006	0.0603	0.0575
0.1	0.1614	0.1345	0.0868	0.0041	0.1544	0.0584	0.0551	0.1547	0.0596	0.0562
0.2	0.3079	0.2805	0.082	0.0041	0.2962	0.0552	0.0527	0.2965	0.0564	0.0538

Table N: Results of $\mu_\alpha = 0.6$, $\sigma_\alpha = 0.2$: K_{YX}

		CD-Ratio			CD-Egger			CD-GLS		
β_{YX}	K_{YX}	Mean($\hat{K}$)	sd($\hat{K}$)	Mean$(se(\hat{K}))$	Mean($\hat{K}$)	sd($\hat{K}$)	Mean$(se(\hat{K}))$	Mean($\hat{K}$)	sd($\hat{K}$)	Mean$(se(\hat{K}))$
-0.2	-0.2535	-0.2754	0.0659	0.0041	-0.2487	0.0448	0.044	-0.2486	0.0449	0.0449
-0.1	-0.129	-0.1595	0.0685	0.0041	-0.1268	0.0463	0.0447	-0.1266	0.0468	0.0456
0	0	-0.0361	0.0706	0.0042	-0.0007	0.0471	0.0447	-0.0005	0.0479	0.0457
0.1	0.1272	0.0901	0.0716	0.0042	0.1236	0.047	0.0441	0.1239	0.048	0.045
0.2	0.2469	0.2122	0.0706	0.0042	0.2408	0.0459	0.0428	0.2411	0.0469	0.0437

Table O: Results of $\mu_\alpha = 1$, $\sigma_\alpha = 0.2$: K_{YX}

		CD-Ratio			CD-Egger			CD-GLS		
β_{YX}	K_{YX}	Mean($\hat{K}$)	sd($\hat{K}$)	Mean$(se(\hat{K}))$	Mean($\hat{K}$)	sd($\hat{K}$)	Mean$(se(\hat{K}))$	Mean($\hat{K}$)	sd($\hat{K}$)	Mean$(se(\hat{K}))$
-0.2	-0.171	-0.2087	0.0464	0.0042	-0.1699	0.031	0.03	-0.1699	0.0312	0.0306
-0.1	-0.0862	-0.1321	0.0471	0.0042	-0.0859	0.0315	0.0302	-0.0858	0.0319	0.0309
0	0	-0.0523	0.0479	0.0043	-0.0005	0.0318	0.0302	-0.0004	0.0324	0.0309
0.1	0.0856	0.0301	0.0486	0.0043	0.0842	0.032	0.03	0.0843	0.0326	0.0307
0.2	0.1687	0.1134	0.0492	0.0043	0.1665	0.0319	0.0296	0.1667	0.0326	0.0303

Goodness-of-Fit Tests

We show the results of the Goodness-of-Fit tests for CD-Ratio, CD-Egger, and CD-GLS. Table P shows the results under setup $\mu_\alpha = 0, \sigma_\alpha = 0$, and various levels of β_{YX}. For each of the three methods, in each of 500 simulations we can calculate the test statistics Q^{YX} and Q^{XY}, then perform the corresponding χ^2-test to get p-value p_{YX} for direction X to Y, and p-value p_{XY} for direction Y to X, respectively. Then we show the proportion out of 500 simulated datasets p_{YX}'s less than 0.05, indicated by "$p_{YX} < 0.05$", and proportion of p_{XY}'s less than 0.05, indicated by "$p_{XY} < 0.05$". Table Q to A4 shows the results for other setups.

Table P: GOF Testing Results of $\mu_\alpha = 0$, $\sigma_\alpha = 0$

	CD-Ratio		CD-Egger		CD-GLS	
β_{YX}	$p_{YX} < 0.05$	$p_{XY} < 0.05$	$p_{YX} < 0.05$	$p_{XY} < 0.05$	$p_{YX} < 0.05$	$p_{XY} < 0.05$
-0.2	0.002	0.012	0	0.014	0	0
-0.1	0.012	0.188	0	0	0	0
0	0.054	0.042	0.002	0	0.002	0.026
0.1	0.022	0.18	0.002	0	0	0
0.2	0	0.022	0	0.006	0	0

Table Q: GOF Results of $\mu_\alpha = 0.2$, $\sigma_\alpha = 0$

	CD-Ratio		CD-Egger		CD-GLS	
β_{YX}	$p_{YX} < 0.05$	$p_{XY} < 0.05$	$p_{YX} < 0.05$	$p_{XY} < 0.05$	$p_{YX} < 0.05$	$p_{XY} < 0.05$
-0.2	1	1	0	0.014	0	0
-0.1	1	1	0	0	0	0
0	1	1	0	0	0	0
0.1	1	1	0	0	0	0
0.2	1	1	0	0.006	0	0

Table R: GOF Results of $\mu_\alpha = 0.4$, $\sigma_\alpha = 0$

	CD-Ratio		CD-Egger		CD-GLS	
β_{YX}	$p_{YX} < 0.05$	$p_{XY} < 0.05$	$p_{YX} < 0.05$	$p_{XY} < 0.05$	$p_{YX} < 0.05$	$p_{XY} < 0.05$
-0.2	1	1	0	0.014	0	0
-0.1	1	1	0	0	0	0
0	1	1	0	0	0	0
0.1	1	1	0	0	0	0
0.2	1	1	0	0.006	0	0

Table S: GOF Results of $\mu_\alpha = 0.6$, $\sigma_\alpha = 0$

	CD-Ratio		CD-Egger		CD-GLS	
β_{YX}	$p_{YX} < 0.05$	$p_{XY} < 0.05$	$p_{YX} < 0.05$	$p_{XY} < 0.05$	$p_{YX} < 0.05$	$p_{XY} < 0.05$
-0.2	1	1	0	0.014	0	0
-0.1	1	1	0	0	0	0
0	1	1	0	0	0	0
0.1	1	1	0	0	0	0
0.2	1	1	0	0.006	0	0

Table T: GOF Results of $\mu_\alpha = 1$, $\sigma_\alpha = 0$

	CD-Ratio		CD-Egger		CD-GLS	
β_{YX}	$p_{YX} < 0.05$	$p_{XY} < 0.05$	$p_{YX} < 0.05$	$p_{XY} < 0.05$	$p_{YX} < 0.05$	$p_{XY} < 0.05$
-0.2	1	1	0	0.014	0	0
-0.1	1	1	0	0	0	0
0	1	1	0	0	0	0
0.1	1	1	0	0	0	0
0.2	1	1	0	0.006	0	0

Table U: GOF Results of $\mu_\alpha = 0$, $\sigma_\alpha = 0.1$

	CD-Ratio		CD-Egger		CD-GLS	
β_{YX}	$p_{YX} < 0.05$	$p_{XY} < 0.05$	$p_{YX} < 0.05$	$p_{XY} < 0.05$	$p_{YX} < 0.05$	$p_{XY} < 0.05$
-0.2	1	1	0	0	0	0
-0.1	1	1	0	0	0	0
0	1	1	0	0	0	0
0.1	1	1	0	0	0	0
0.2	1	1	0	0	0	0

Table V: GOF Results of $\mu_\alpha = 0.2$, $\sigma_\alpha = 0.1$

	CD-Ratio		CD-Egger		CD-GLS	
β_{YX}	$p_{YX} < 0.05$	$p_{XY} < 0.05$	$p_{YX} < 0.05$	$p_{XY} < 0.05$	$p_{YX} < 0.05$	$p_{XY} < 0.05$
-0.2	1	1	0	0	0	0
-0.1	1	1	0	0	0	0
0	1	1	0	0	0	0
0.1	1	1	0	0	0	0
0.2	1	1	0	0	0	0

Table W: GOF Results of $\mu_\alpha = 0.4$, $\sigma_\alpha = 0.1$

	CD-Ratio		CD-Egger		CD-GLS	
β_{YX}	$p_{YX} < 0.05$	$p_{XY} < 0.05$	$p_{YX} < 0.05$	$p_{XY} < 0.05$	$p_{YX} < 0.05$	$p_{XY} < 0.05$
-0.2	1	1	0	0	0	0
-0.1	1	1	0	0	0	0
0	1	1	0	0	0	0
0.1	1	1	0	0	0	0
0.2	1	1	0	0	0	0

Table X: GOF Results of $\mu_\alpha = 0.6$, $\sigma_\alpha = 0.1$

	CD-Ratio		CD-Egger		CD-GLS	
β_{YX}	$p_{YX} < 0.05$	$p_{XY} < 0.05$	$p_{YX} < 0.05$	$p_{XY} < 0.05$	$p_{YX} < 0.05$	$p_{XY} < 0.05$
-0.2	1	1	0	0	0	0
-0.1	1	1	0	0	0	0
0	1	1	0	0	0	0
0.1	1	1	0	0	0	0
0.2	1	1	0	0	0	0

Table Y: GOF Results of $\mu_\alpha = 1$, $\sigma_\alpha = 0.1$

	CD-Ratio		CD-Egger		CD-GLS	
β_{YX}	$p_{YX} < 0.05$	$p_{XY} < 0.05$	$p_{YX} < 0.05$	$p_{XY} < 0.05$	$p_{YX} < 0.05$	$p_{XY} < 0.05$
-0.2	1	1	0	0	0	0
-0.1	1	1	0	0	0	0
0	1	1	0	0	0	0
0.1	1	1	0	0	0	0
0.2	1	1	0	0	0	0

Table Z: GOF Results of $\mu_\alpha = 0$, $\sigma_\alpha = 0.2$

	CD-Ratio		CD-Egger		CD-GLS	
β_{YX}	$p_{YX} < 0.05$	$p_{XY} < 0.05$	$p_{YX} < 0.05$	$p_{XY} < 0.05$	$p_{YX} < 0.05$	$p_{XY} < 0.05$
-0.2	1	1	0	0	0	0
-0.1	1	1	0	0	0	0
0	1	1	0	0	0	0
0.1	1	1	0	0	0	0
0.2	1	1	0	0	0	0

Table A1: GOF Results of $\mu_\alpha = 0.2$, $\sigma_\alpha = 0.2$

	CD-Ratio		CD-Egger		CD-GLS	
β_{YX}	$p_{YX} < 0.05$	$p_{XY} < 0.05$	$p_{YX} < 0.05$	$p_{XY} < 0.05$	$p_{YX} < 0.05$	$p_{XY} < 0.05$
-0.2	1	1	0	0	0	0
-0.1	1	1	0	0	0	0
0	1	1	0	0	0	0
0.1	1	1	0	0	0	0
0.2	1	1	0	0	0	0

Table A2: GOF Results of $\mu_\alpha = 0.4$, $\sigma_\alpha = 0.2$

	CD-Ratio		CD-Egger		CD-GLS	
β_{YX}	$p_{YX} < 0.05$	$p_{XY} < 0.05$	$p_{YX} < 0.05$	$p_{XY} < 0.05$	$p_{YX} < 0.05$	$p_{XY} < 0.05$
-0.2	1	1	0	0	0	0
-0.1	1	1	0	0	0	0
0	1	1	0	0	0	0
0.1	1	1	0	0	0	0
0.2	1	1	0	0	0	0

Table A3: GOF Results of $\mu_\alpha = 0.6$, $\sigma_\alpha = 0.2$

β_{YX}	CD-Ratio		CD-Egger		CD-GLS	
	$p_{YX} < 0.05$	$p_{XY} < 0.05$	$p_{YX} < 0.05$	$p_{XY} < 0.05$	$p_{YX} < 0.05$	$p_{XY} < 0.05$
-0.2	1	1	0	0	0	0
-0.1	1	1	0	0	0	0
0	1	1	0	0	0	0
0.1	1	1	0	0	0	0
0.2	1	1	0	0	0	0

Table A4: GOF Results of $\mu_\alpha = 1$, $\sigma_\alpha = 0.2$

β_{YX}	CD-Ratio		CD-Egger		CD-GLS	
	$p_{YX} < 0.05$	$p_{XY} < 0.05$	$p_{YX} < 0.05$	$p_{XY} < 0.05$	$p_{YX} < 0.05$	$p_{XY} < 0.05$
-0.2	1	1	0	0	0	0
-0.1	1	1	0	0	0	0
0	1	1	0	0	0	0
0.1	1	1	0	0	0	0
0.2	1	1	0	0	0	0

Comparing the Decisions

We compare CD-Ratio, CD-Egger and CD-GLS with MR Steiger's and bi-directional MR in terms of their decisions made on the causal direction. Similar to Steiger's method, we also applied CD-Ratio to each of the 22 SNPs, then calculated the proportions of the three possible decisions, denoted by "CD-Ratio-Prop". We also pooled together the individual results from each of the 22 SNPs by majority voting to reach a final decision, denoted as "CD-Ratio-MV". For other methods, their notations are the same as in Fig. 9 in the main text. Figure A shows the comparison results for setup $\beta_{YX} = 0, \mu_\alpha = 0$, and $\sigma_\alpha = 0, 0.1, 0.2$. Figure B to Y show the comparison results for other simulation setups.

Figure A: Relative frequencies of decisions for causal direction of all methods: $\beta_{YX} = 0$, $\mu_\alpha = 0$

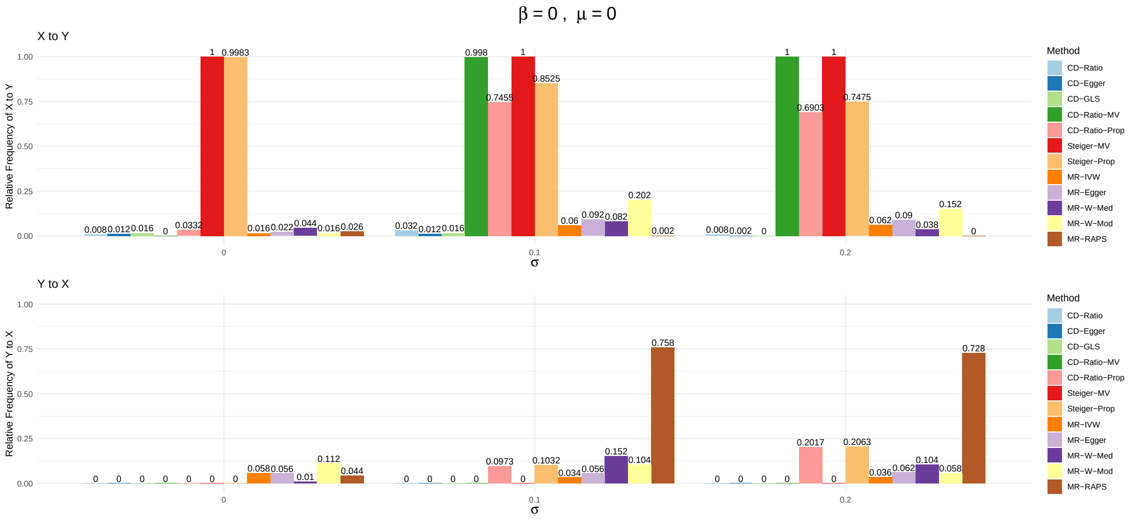

Figure B: Relative frequencies of decisions for causal direction of all methods: $\beta_{YX} = 0$, $\mu_{\alpha} = 0.2$

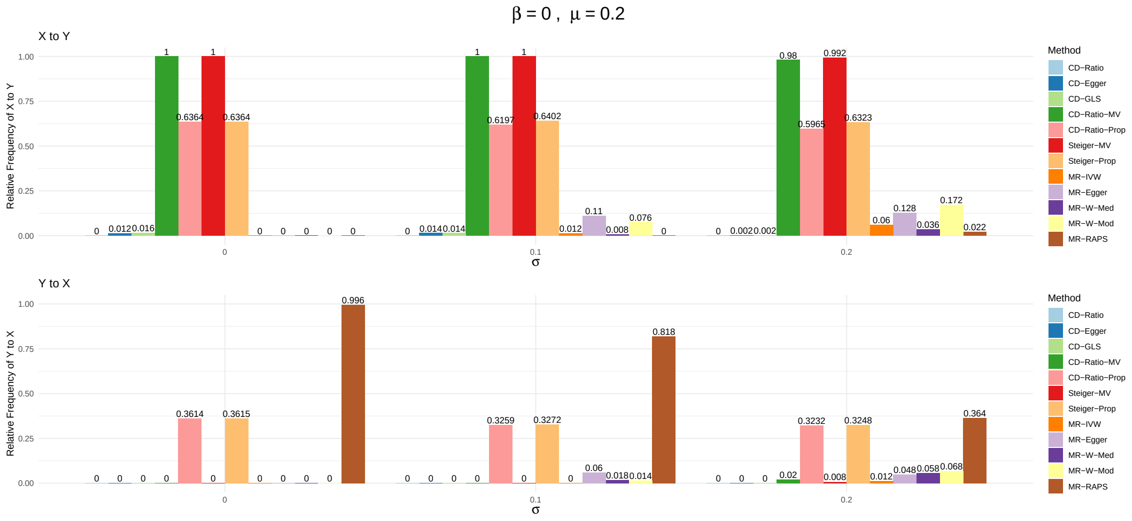

Figure C: Relative frequencies of decisions for causal direction of all methods: $\beta_{YX} = 0$, $\mu_{\alpha} = 0.4$

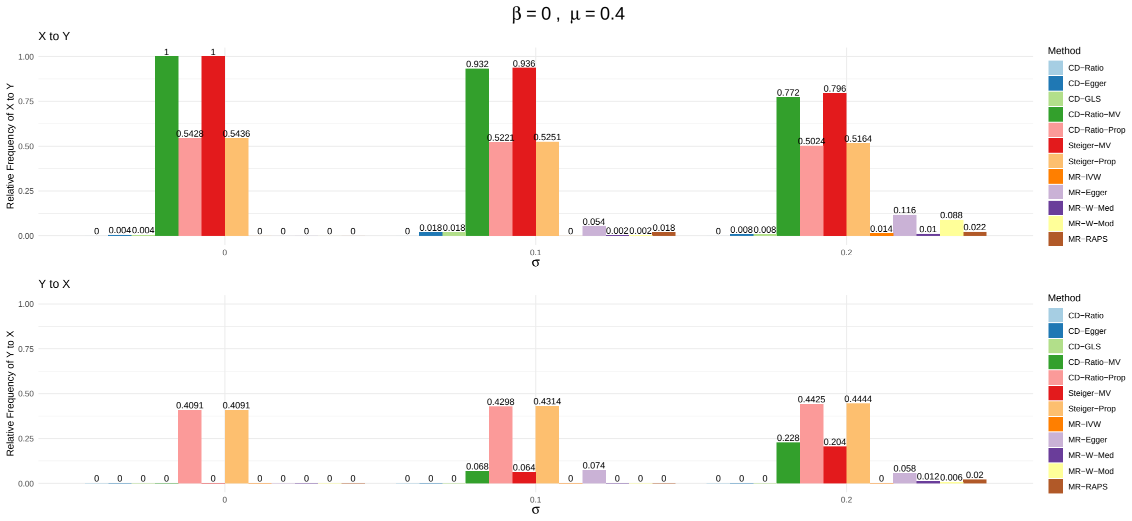

Figure D: Relative frequencies of decisions for causal direction of all methods: $\beta_{YX} = 0$, $\mu_\alpha = 0.6$

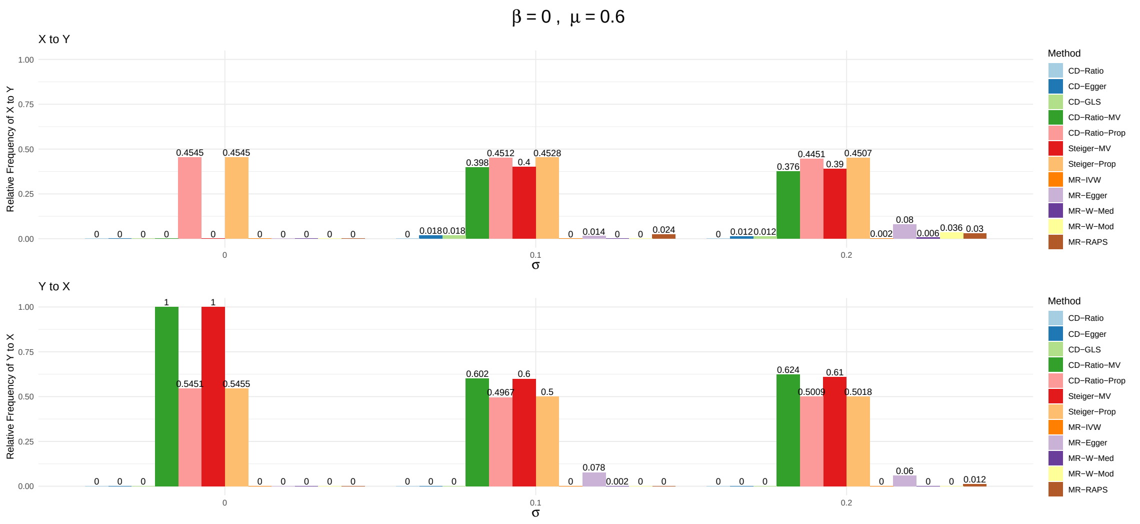

Figure E: Relative frequencies of decisions for causal direction of all methods: $\beta_{YX} = 0$, $\mu_\alpha = 1$

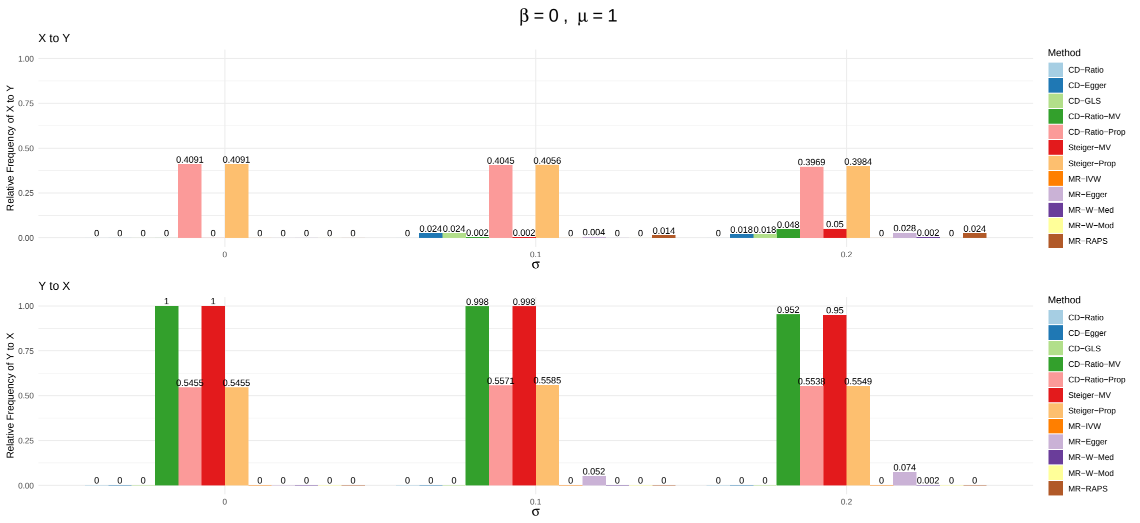

Figure F: Relative frequencies of decisions for causal direction of all methods: $\beta_{YX} = 0.1$, $\mu_\alpha = 0$

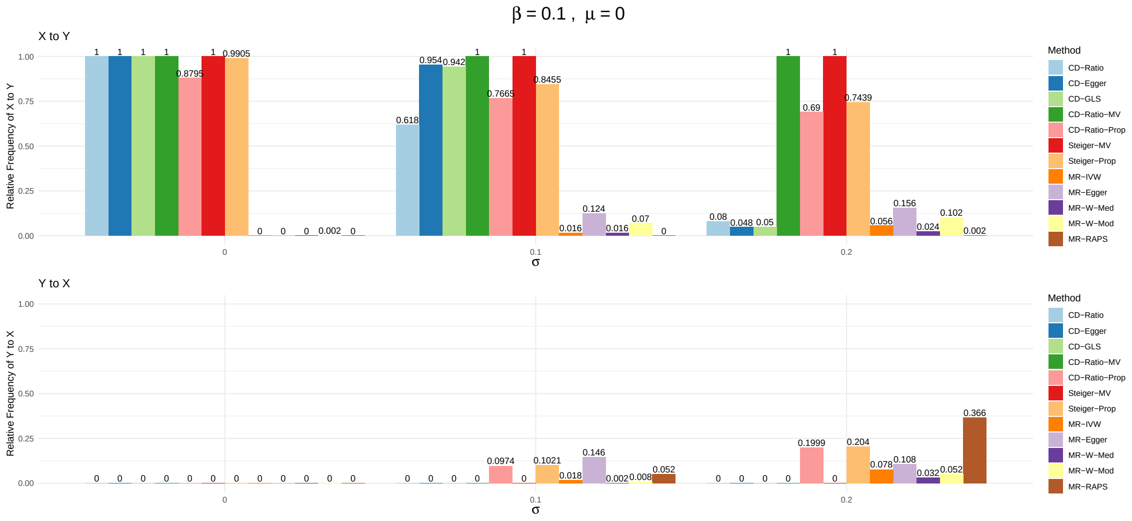

Figure G: Relative frequencies of decisions for causal direction of all methods: $\beta_{YX} = 0.1$, $\mu_\alpha = 0.2$

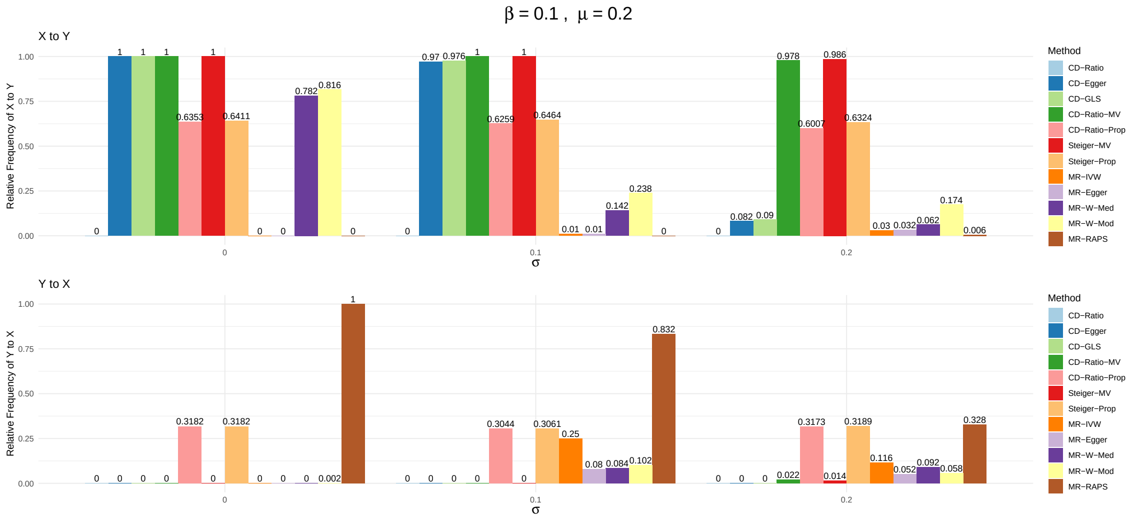

Figure H: Relative frequencies of decisions for causal direction of all methods: $\beta_{YX} = 0.1$, $\mu_\alpha = 0.4$

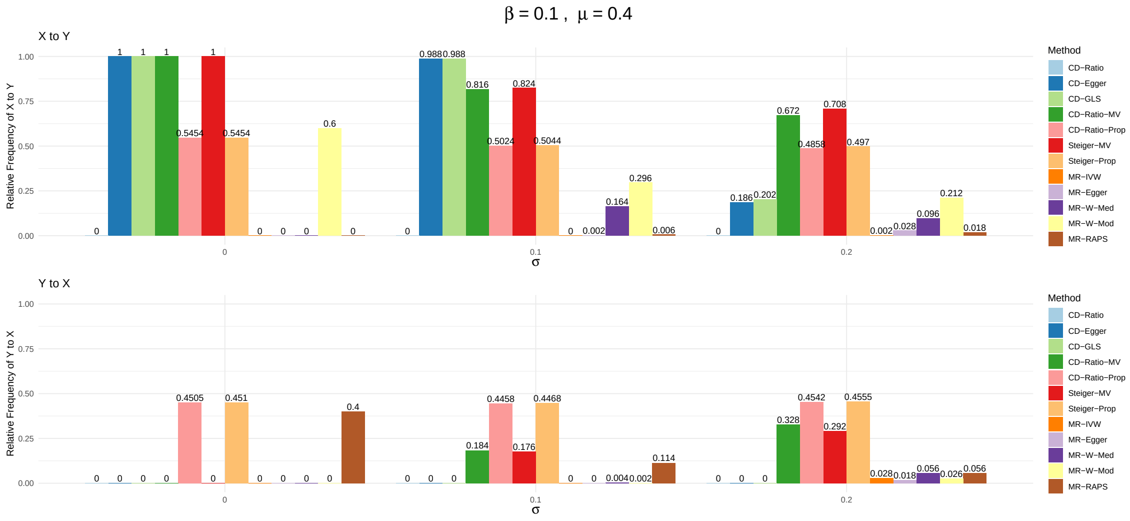

Figure I: Relative frequencies of decisions for causal direction of all methods: $\beta_{YX} = 0.1$, $\mu_\alpha = 0.6$

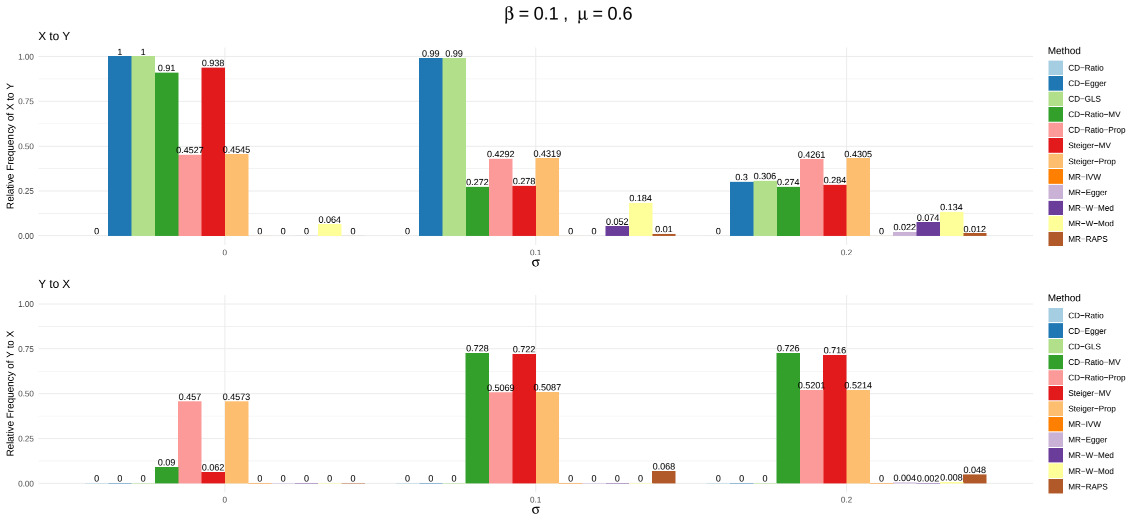

Figure J: Relative frequencies of decisions for causal direction of all methods: $\beta_{YX} = 0.1$, $\mu_\alpha = 1$

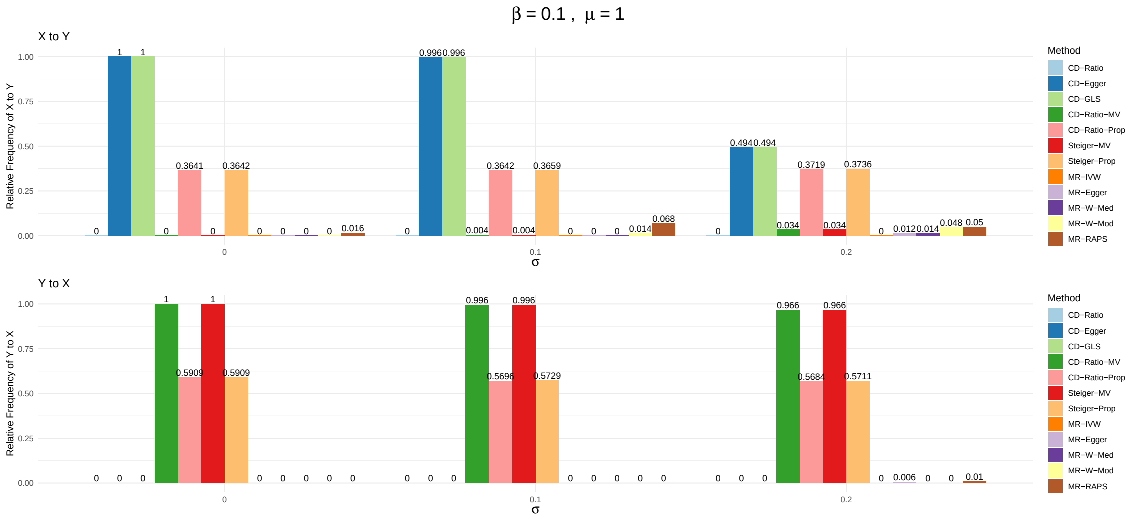

Figure K: Relative frequencies of decisions for causal direction of all methods: $\beta_{YX} = -0.1$, $\mu_\alpha = 0$

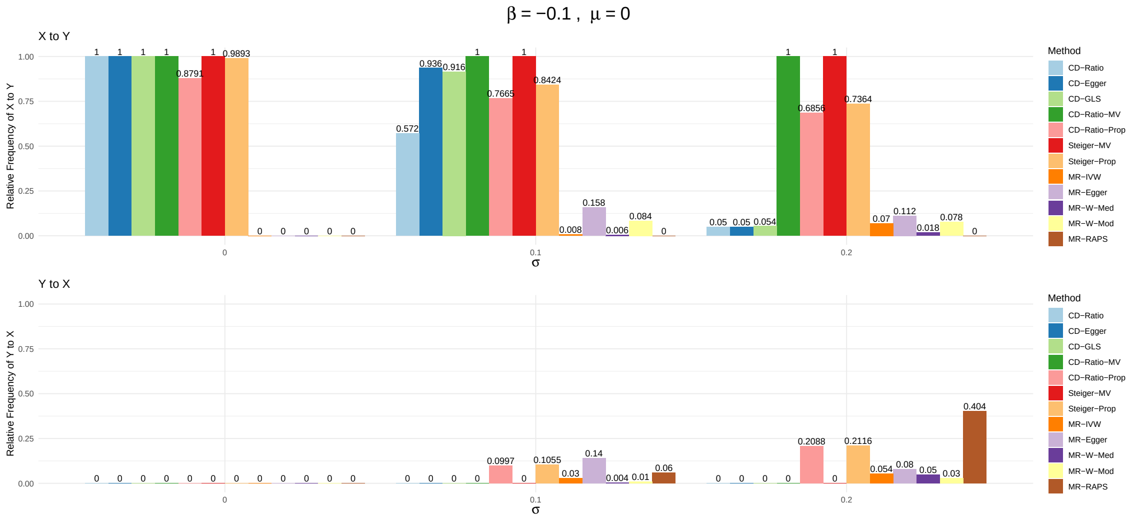

Figure L: Relative frequencies of decisions for causal direction of all methods: $\beta_{YX} = -0.1$, $\mu_\alpha = 0.2$

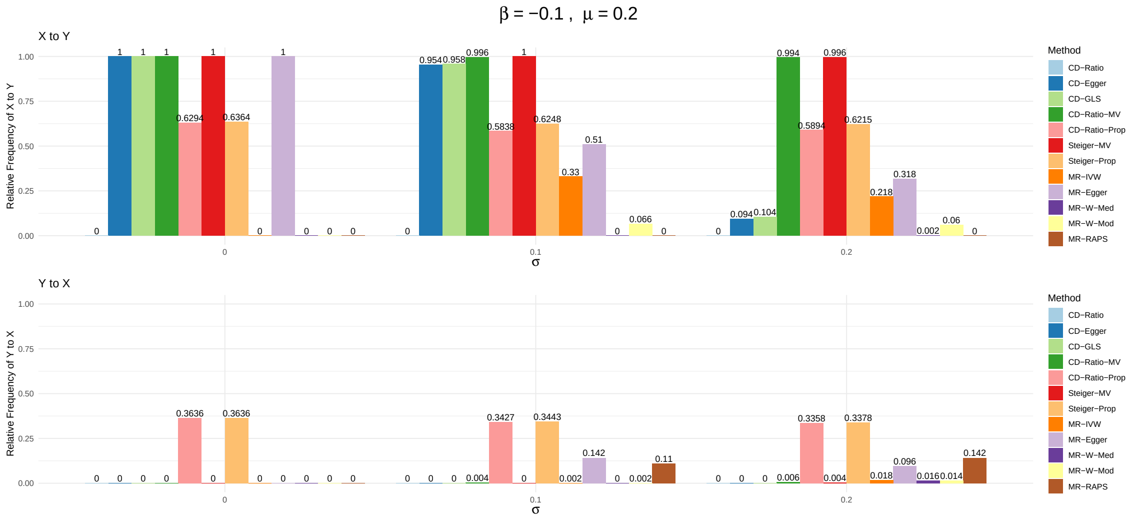

Figure M: Relative frequencies of decisions for causal direction of all methods: $\beta_{YX} = -0.1$, $\mu_\alpha = 0.4$

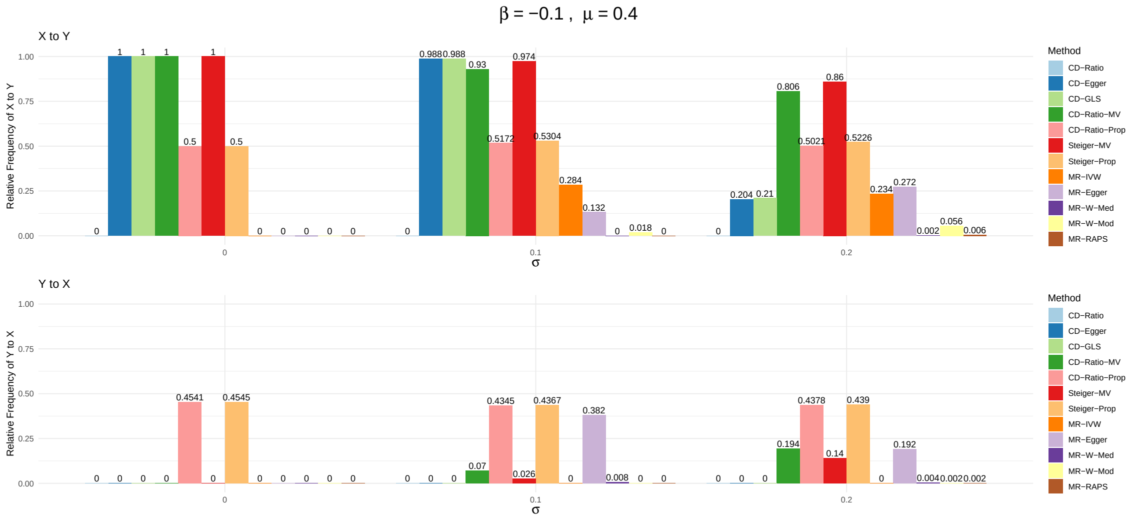

Figure N: Relative frequencies of decisions for causal direction of all methods: $\beta_{YX} = -0.1$, $\mu_\alpha = 0.6$

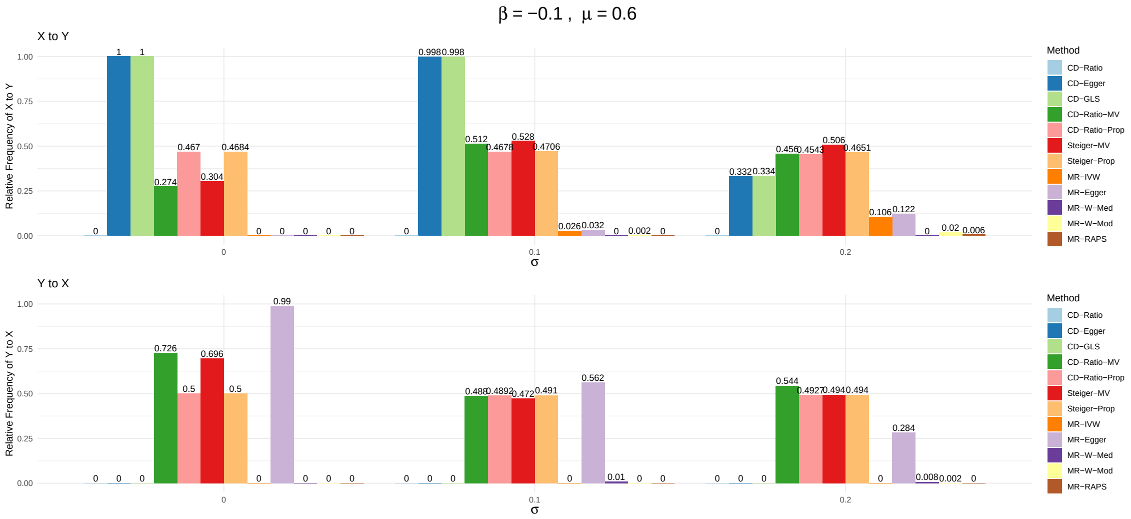

Figure O: Relative frequencies of decisions for causal direction of all methods: $\beta_{YX} = -0.1$, $\mu_\alpha = 1$

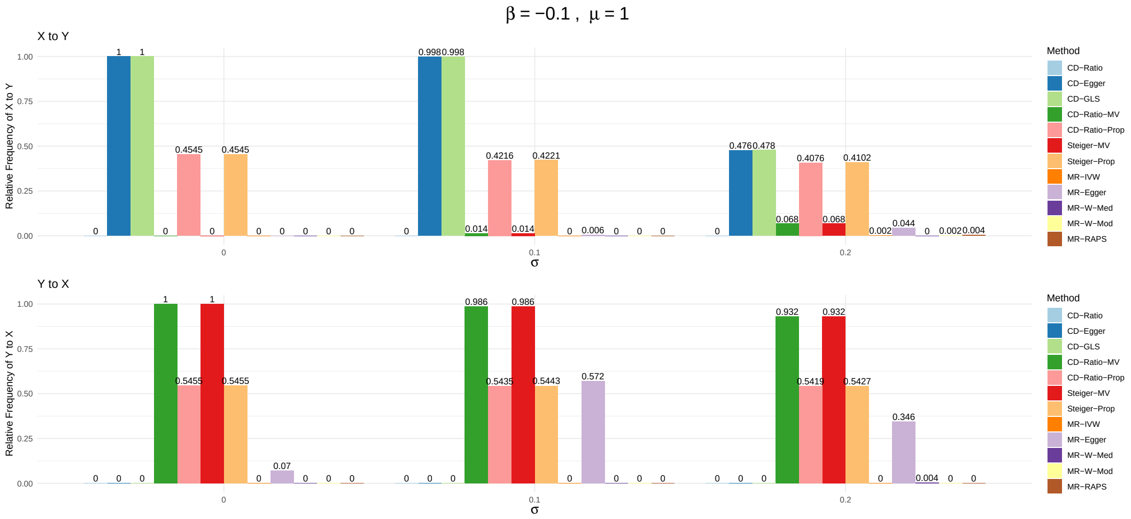

Figure P: Relative frequencies of decisions for causal direction of all methods: $\beta_{YX} = 0.2$, $\mu_{\alpha} = 0$

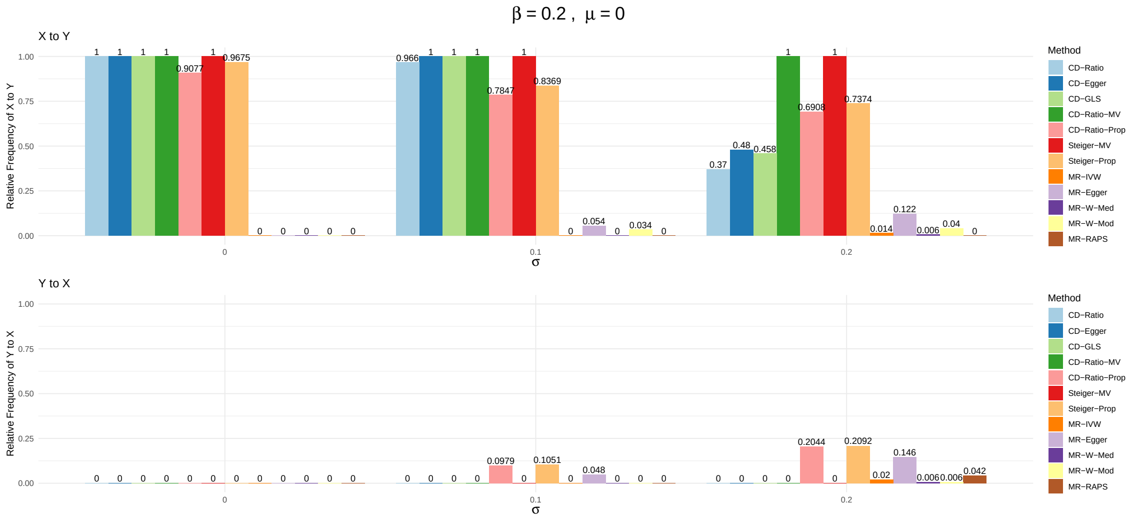

Figure Q: Relative frequencies of decisions for causal direction of all methods: $\beta_{YX} = 0.2$, $\mu_{\alpha} = 0.2$

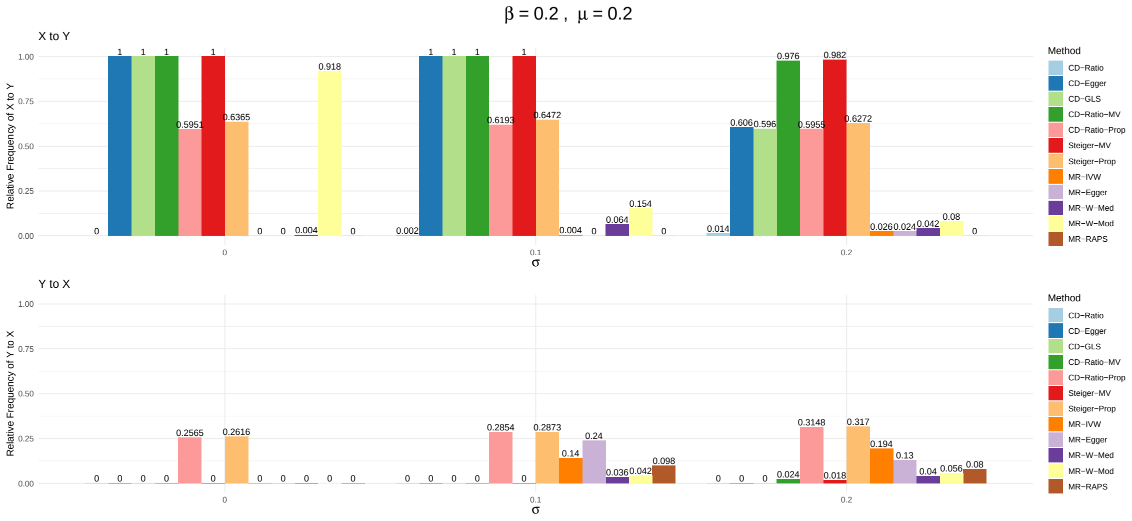

Figure R: Relative frequencies of decisions for causal direction of all methods: $\beta_{YX} = 0.2$, $\mu_\alpha = 0.4$

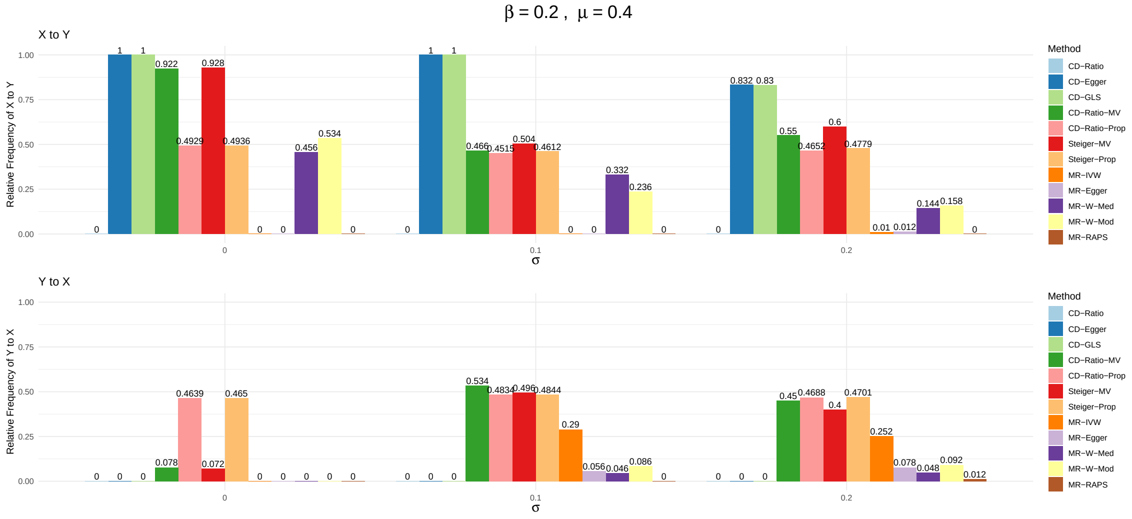

Figure S: Relative frequencies of decisions for causal direction of all methods: $\beta_{YX} = 0.2$, $\mu_\alpha = 0.6$

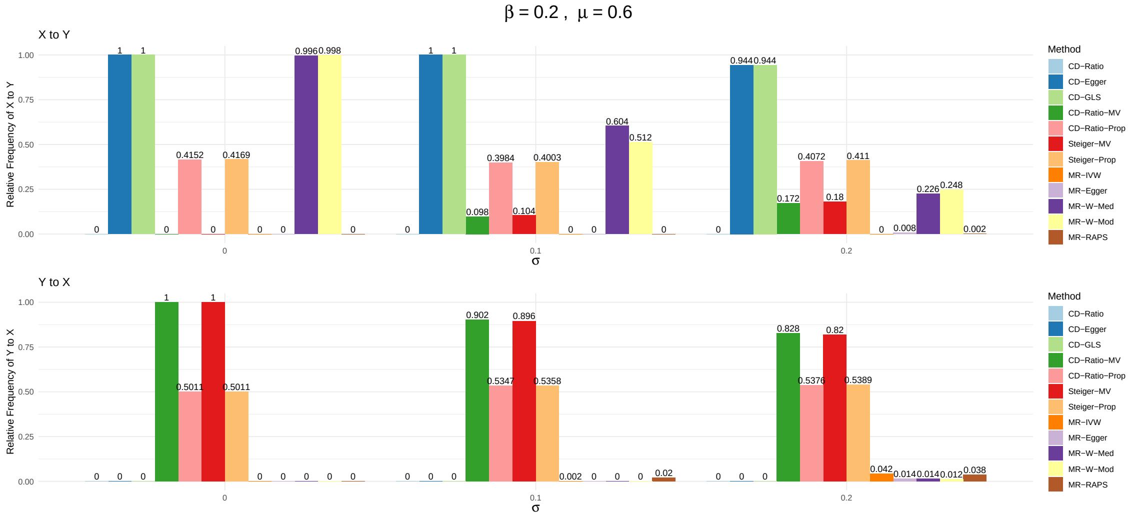

Figure T: Relative frequencies of decisions for causal direction of all methods: $\beta_{YX} = 0.2$, $\mu_\alpha = 1$

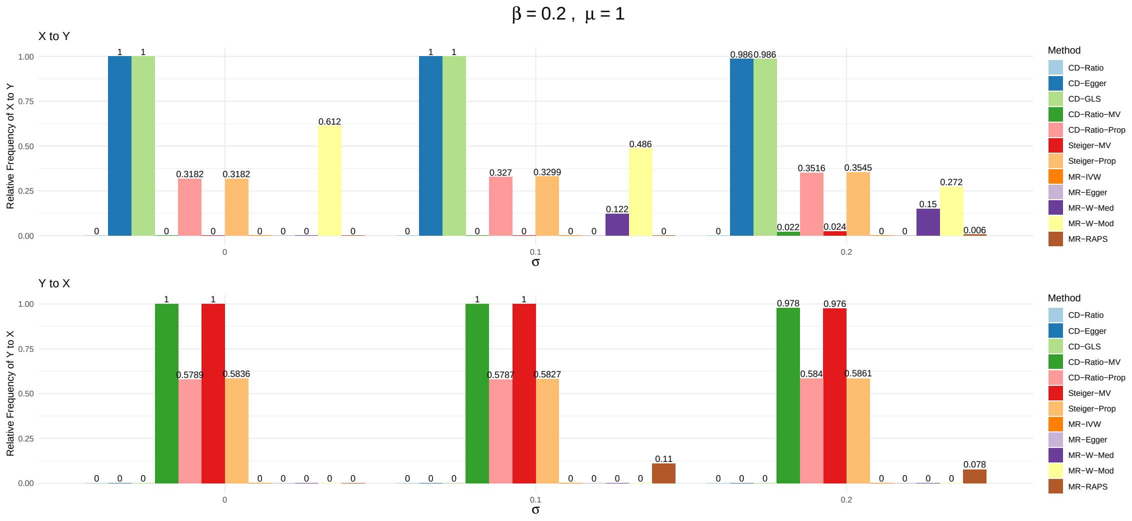

Figure U: Relative frequencies of decisions for causal direction of all methods: $\beta_{YX} = -0.2$, $\mu_\alpha = 0$

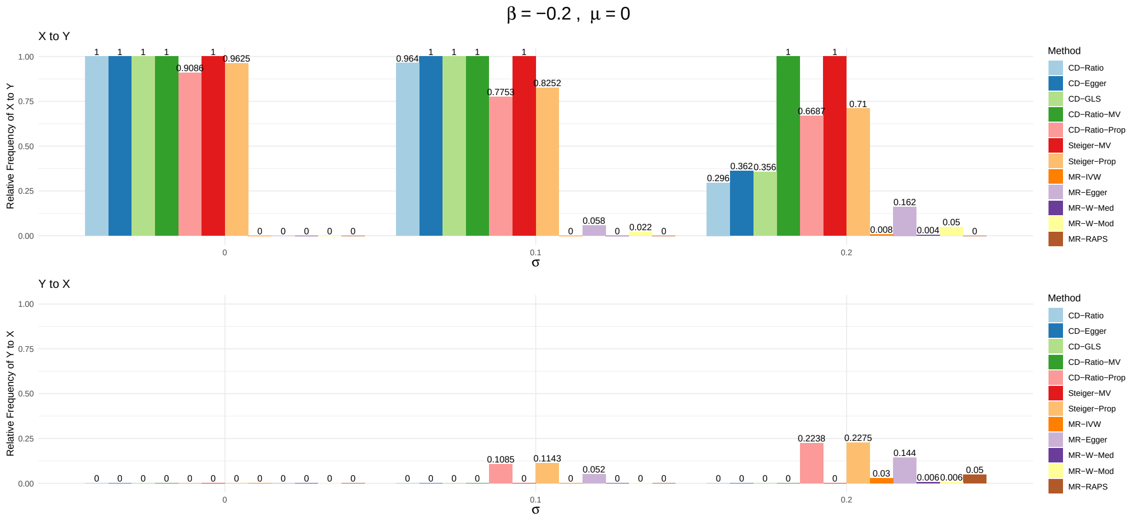

Figure V: Relative frequencies of decisions for causal direction of all methods: $\beta_{YX} = -0.2$, $\mu_\alpha = 0.2$

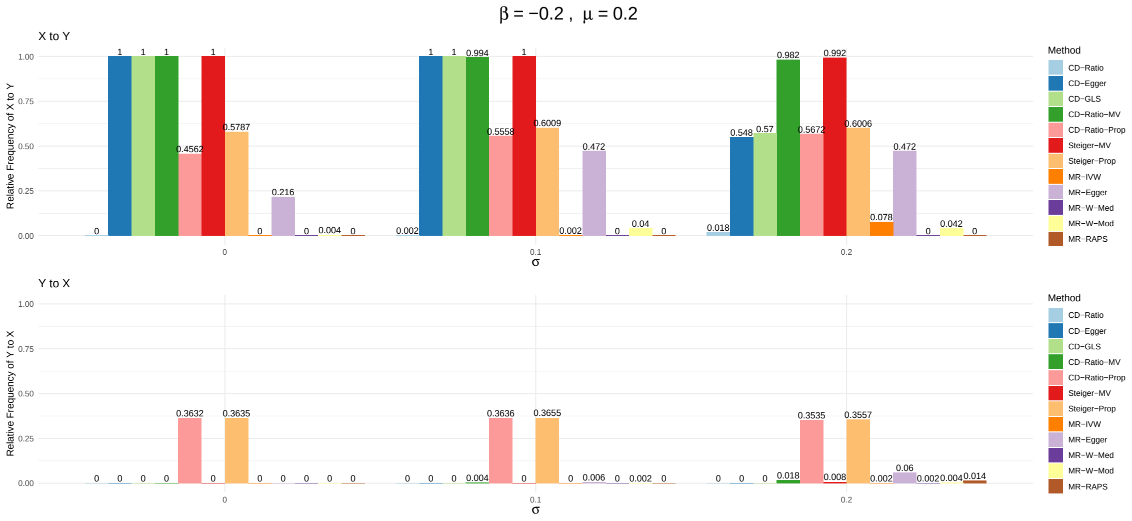

Figure W: Relative frequencies of decisions for causal direction of all methods: $\beta_{YX} = -0.2$, $\mu_\alpha = 0.4$

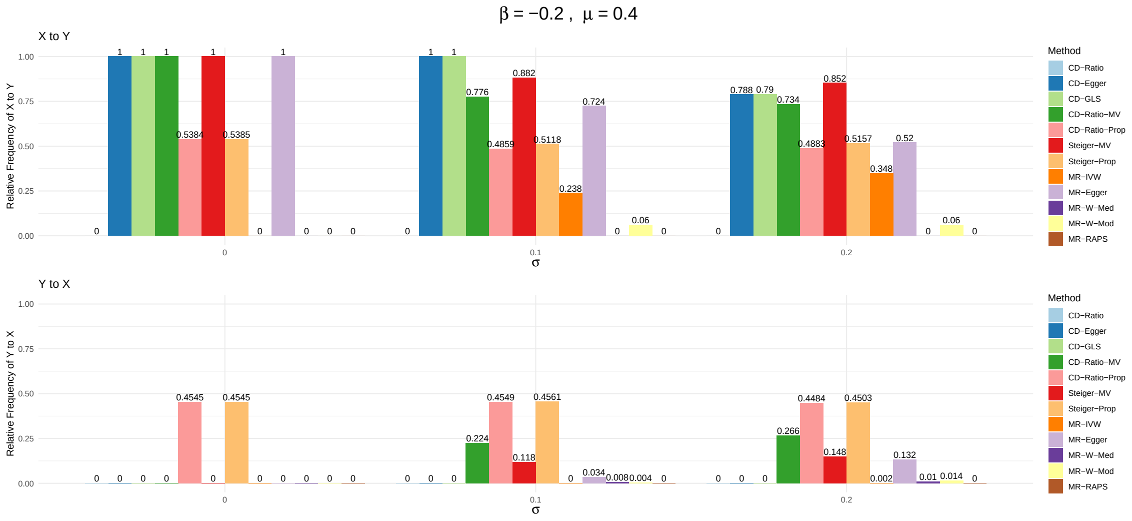

Figure X: Relative frequencies of decisions for causal direction of all methods: $\beta_{YX} = -0.2$, $\mu_\alpha = 0.6$

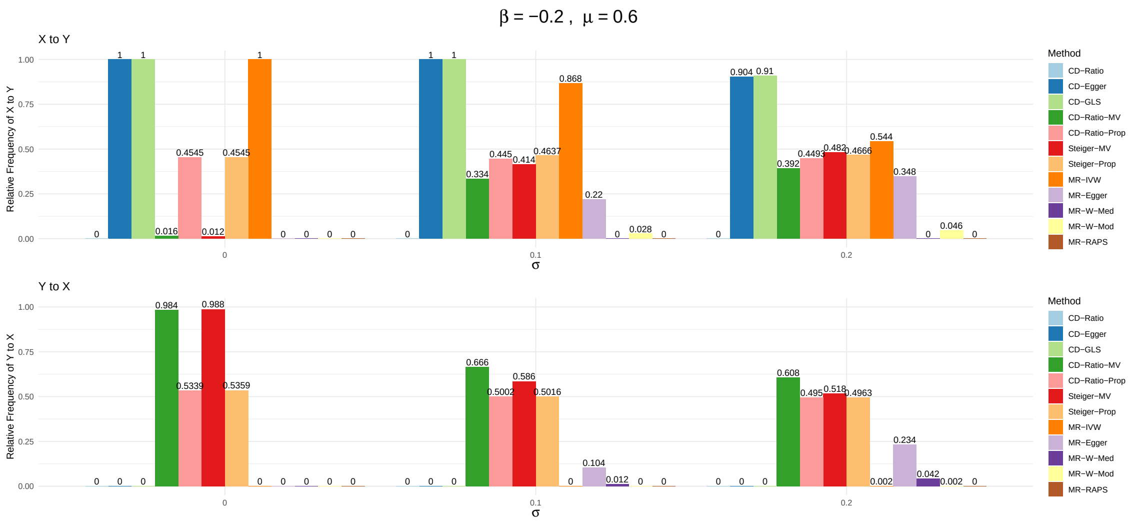

Figure Y: Relative frequencies of decisions for causal direction of all methods: $\beta_{YX} = -0.2$, $\mu_\alpha = 1$

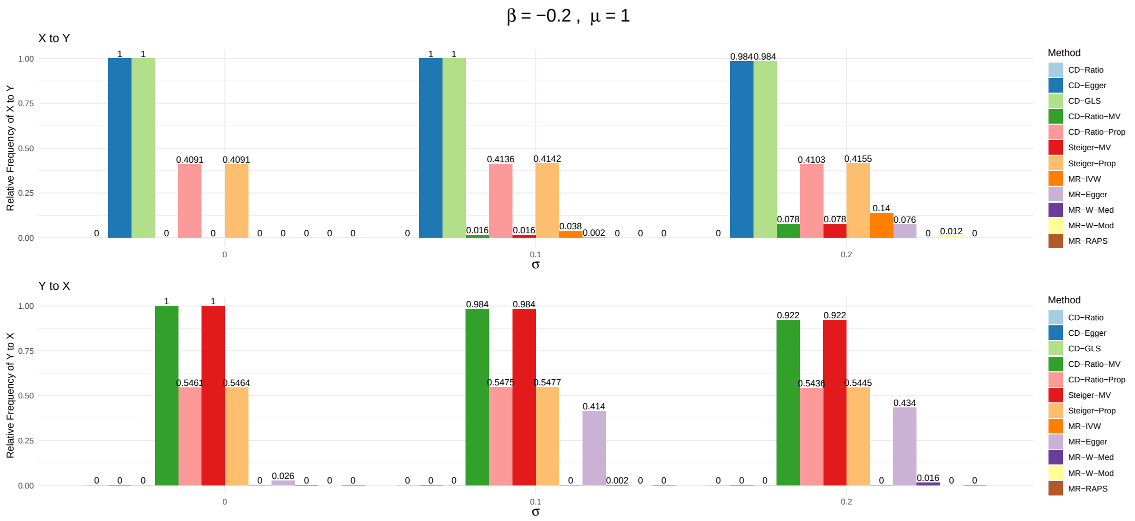

Supplement: S6 Text — (PDF) [file pgen.1009105.s006.pdf]
