# Supplementary material for: Inferring causal direction between two traits in the presence of horizontal pleiotropy with GWAS summary data
Source: PLoS Genet. 2020 Nov 2;16(11):e1009105. doi: 10.1371/journal.pgen.1009105 (PMC7660933; doi:10.1371/journal.pgen.1009105)
Supplement: S7 Text — (PDF) [file pgen.1009105.s007.pdf]

S7 Text: Simulations for Bi-directional Causal Effects

We set the sample size $n = 10000$, generated g_1 and g_2 independently with a minor allele frequency of 0.3. We generated the independent error terms ε_1 and ε_2 from a normal distribution with mean 0 and variance 3.2, and generated the confounder U from a normal distribution with mean 0 and variance 0.8. We set $\gamma_1 = 1$, $\gamma_2 = 1$, $\beta_{XU} = 1$, and $\beta_{YU} = 1$. With different β_1 and β_2, we generated two independent samples of X and Y from the reduced form of the models (25) in the main text, using the first sample to get summary statistics for X and the second for Y, and applied **CD-Ratio** to both directions, leading to out **bi-CD-Ratio**. For comparison, we also applied **MR-Steiger** and **MR-Wald-Ratio** to both directions. When considering the candidate direction of X to Y, we used g_1 as the IV; for Y to X, we used g_2 as the IV. For each setup we did simulations 1000 times, and drew conclusions on both directions based on the 95% confidence intervals. Table A shows the simulation results of estimating K_1, K_2 and their standard errors with bi-CD-Ratio, and Table B compares *bi-directional) CD-Ratio, MR-Steiger, and MR-Wald-Ratio for their relative frequencies of concluding with any causal directions.

Table A: Simulation results for bi-CD-Ratio with different pairs of β_1 and β_2. For both directions we show the true value of K, the mean and standard deviation of estimates, and mean of standard errors.

No causal effect								
	$X \to Y$				$Y \to X$			
(β_1, β_2)	K_1	Mean($\hat{K}_1$)	sd($\hat{K}_1$)	Mean($se(\hat{K}_1)$)	K_2	Mean($\hat{K}_2$)	sd($\hat{K}_2$)	Mean($se(\hat{K}_2)$)
(0,0)	0	0.001	0.032	0.033	0	0.001	0.032	0.032
Unidirectional causal effect from X to Y								
	$X \to Y$				$Y \to X$			
(β_1, β_2)	K_1	Mean($\hat{K}_1$)	sd($\hat{K}_1$)	Mean($se(\hat{K}_1)$)	K_2	Mean($\hat{K}_2$)	sd($\hat{K}_2$)	Mean($se(\hat{K}_2)$)
(-0.2,0)	-0.203	-0.203	0.032	0.033	0	0.001	0.032	0.032
(0.2,0)	0.19	0.191	0.032	0.033	0	0.001	0.034	0.034
Unidirectional causal effect from Y to X								
	$X \to Y$				$Y \to X$			
(β_1, β_2)	K_1	Mean($\hat{K}_1$)	sd($\hat{K}_1$)	Mean($se(\hat{K}_1)$)	K_2	Mean($\hat{K}_2$)	sd($\hat{K}_2$)	Mean($se(\hat{K}_2)$)
(0,-0.2)	0	0.001	0.031	0.032	-0.203	-0.203	0.033	0.033
(0,0.2)	0	0.001	0.034	0.034	0.19	0.191	0.032	0.033
Bi-directional causal effect								
	$X \to Y$				$Y \to X$			
(β_1, β_2)	K_1	Mean($\hat{K}_1$)	sd($\hat{K}_1$)	Mean($se(\hat{K}_1)$)	K_2	Mean($\hat{K}_2$)	sd($\hat{K}_2$)	Mean($se(\hat{K}_2)$)
(-0.2,-0.2)	-0.2	-0.199	0.031	0.032	-0.2	-0.199	0.032	0.032
(-0.2,0.2)	-0.214	-0.214	0.034	0.035	0.187	0.188	0.032	0.032
(0.2, -0.2)	0.187	0.188	0.032	0.032	-0.214	-0.214	0.035	0.035
(0.2, 0.2)	0.2	0.202	0.034	0.035	0.2	0.201	0.034	0.035

Table B: Comparison of (bi-directional) CD-Ratio, MR-Steiger and MR-Wald-Ratio for the relative frequencies of their conclusions on the causal directions.

No causal effect						
	$X \rightarrow Y$			$Y \rightarrow X$		
(β_1, β_2)	CD-Ratio	MR-Steiger	MR-Wald-Ratio	CD-Ratio	MR-Steiger	MR-Wald-Ratio
(0,0)	0.035	1	0.035	0.047	1	0.047
Unidirectional causal effect from X to Y						
	$X \rightarrow Y$			$Y \rightarrow X$		
(β_1, β_2)	CD-Ratio	MR-Steiger	MR-Wald-Ratio	CD-Ratio	MR-Steiger	MR-Wald-Ratio
(-0.2,0)	1	1	1	0.047	1	0.047
(0.2,0)	1	1	1	0.047	1	0.047
Unidirectional causal effect from Y to X						
	$X \rightarrow Y$			$Y \rightarrow X$		
(β_1, β_2)	CD-Ratio	MR-Steiger	MR-Wald-Ratio	CD-Ratio	MR-Steiger	MR-Wald-Ratio
(0,-0.2)	0.035	1	0.035	1	1	1
(0,0.2)	0.035	1	0.035	0.999	1	0.999
Bi-directional causal effect						
	$X \rightarrow Y$			$Y \rightarrow X$		
(β_1, β_2)	CD-Ratio	MR-Steiger	MR-Wald-Ratio	CD-Ratio	MR-Steiger	MR-Wald-Ratio
(-0.2,-0.2)	1	1	1	1	1	1
(-0.2,0.2)	1	1	1	0.999	1	0.999
(0.2,-0.2)	1	1	1	1	1	1
(0.2,0.2)	1	1	1	0.999	1	0.999

From Table A, we can see that for all situations, our proposed bi-CD-Ratio could estimate the true K_1 and K_2 pretty well, and the means of $\text{se}(\hat{K}_1)$ and $\text{se}(\hat{K}_1)$ were close to $\text{sd}(\hat{K}_1)$ and $\text{sd}(\hat{K}_2)$. From Table B, when there was no causal relationship, both the bi-CD-Ratio and MR-Wald-Ratio could control the Type-I Errors around 0.05; when there was a causal direction, both methods could always detect it with a relative frequency of 1. MR-Steiger always concluded with the bi-directional causal effect due to the following reason: for X to Y we used g_1 as the valid instrument; g_1 always had a larger correlation with X than that with Y no matter whether X had a causal effect on Y or not; hence Steger's method would always conclude with a causal direction from X to Y.; similarly, when considering Y to X with g_2 as the instrument, it would always conclude with a causal direction of Y to X. and same for Y to X. In contrast, based on correlation ratios, (bi-directional) CD-Ratio could determine the existence of a causal effect correctly by comparing the ratio with 0; on the other hand, MR-Steiger, based on differences of correlations, could not correctly determine the existence of a causal relationship under this situation.
