# Supplementary material for: Inferring causal direction between two traits in the presence of horizontal pleiotropy with GWAS summary data
Source: PLoS Genet. 2020 Nov 2;16(11):e1009105. doi: 10.1371/journal.pgen.1009105 (PMC7660933; doi:10.1371/journal.pgen.1009105)
Supplement: S8 Text — (PDF) [file pgen.1009105.s008.pdf]

# S8 Text: Simulations for TWAS with Small Sample Sizes

We did a simulation study to investigate how the asymptotic theory for the sample correlations and their ratios performs with smaller sample sizes as typical with molecular endophenotypes as in TWAS. To mimick real TWAS, we used the fitted model from the ADNI gene expression and genotype data for 712 individuals as the data-generating model. For gene **PSPH** on chromosome 7, we identified 10 *SNP*s collectively explaining around 20% of the gene's expression variation in the fitted linear regression model: rs35515795, rs79278832, rs12154781, rs7791829, rs1057603, rs148444659, rs2242509, rs2908543, rs816417 and rs56875346. Figure A shows the correlation matrix of these 10 SNPs based on 712 individuals. From the fitted the linear regression model, we obtained the estimated regression coefficients $\hat{\beta}$'s for these 10 SNPs, and the estimated standard deviation of the error term $\hat{\sigma} = 0.803$. We used $\hat{\beta}$'s and $\hat{\sigma}$ in the corresponding linear model to generate realistic simulated data.

Figure A: Correlation Structure of 10 SNPs in gene PSPH

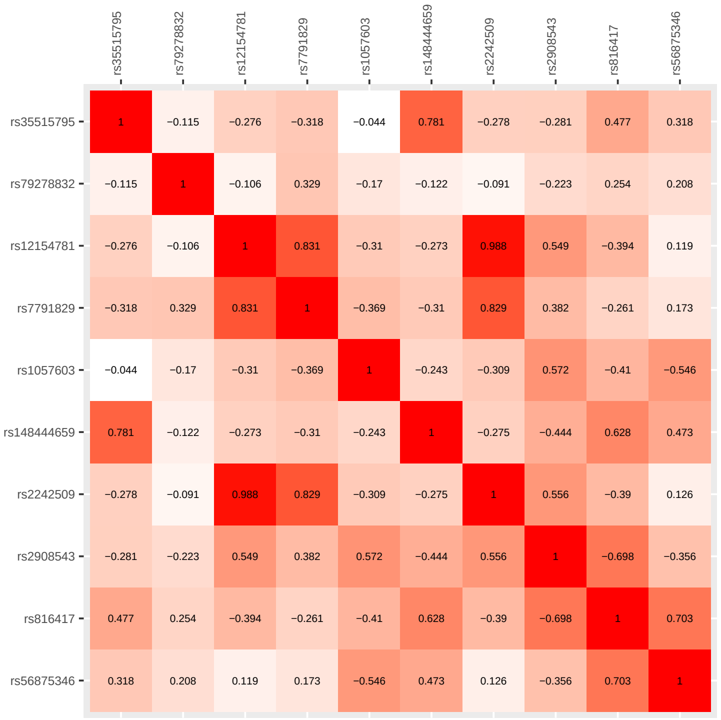


We generated the simulated data as the following:

$$
\begin{aligned}
&U \sim N(0, \hat{\sigma}^2/4) \\
&X = \sum_{j=1}^{10} \hat{\beta}_j \cdot SNP_j + U + \varepsilon_X, \varepsilon_X \sim N(0, \hat{\sigma}^2) \\
&Y = \beta_{YX} \cdot X + U + \varepsilon_Y, \varepsilon_Y \sim N(0, \hat{\sigma}^2)
\end{aligned}
\tag{1}
$$

Here $U$ is a confounder. We generated two independent samples. For the first sample, we randomly chose 200 out of the 712 ADNI individuals, and duplicated their genotypes $k \geq 1$ times to possibly increase the sample size. For the second sample, we randomly chose 150 from the remaining 512 individuals, and duplicated their genotypes $k$ times. With the first sample we obtains the sample correlations between $X$ and the 10 SNPs, and with the second sample the correlations between $Y$ and the 10 SNPs. Then we applied our CD-Ratio method to the two samples of these correlations. We applied CD-Ratio

to each of 10 SNPs, and to combine their results; we also applied it to combine only those of the 9 SNPs without rs2242509, which had a high correlation $> 0.9$ with rs12154781. For each combination of $k = 1,2,3,4,5$ and $\beta_{YX} = -0.2, -0.1, 0, 0.2, 0.1$, we did simulations 1000 times. Each time we got a 95% CI for $K_{YX}$, then if it was completely inside [-1,0) or (0,1], we concluded that $X$ had a causal effect on $Y$; if it covered 0, we concluded that $X$ had no causal effect on $Y$. We also apply the MR Steiger method with each single SNP for comparison. Note that here, for simplicity, differing from our other numerical studies, we only considered either no or one causal direction from $X$ to $Y$. Table A shows the simulation results.

Table A: Relative frequencies of concluding with $X$ having a causal effect on $Y$ from 1000 simulations for each setup of $(\beta_{YX}, k)$. For CD-Ratio, the results combining over 10 or 9 SNPs, and the maximum (max), mean and minimum (min) relative frequencies using each of the 10 single SNPs are shown. For MR-Steiger, the maximum, mean and minimum of relative frequencies using each of 10 SNPs are shown.

| $\beta_{YX} = 0$ | | | | | | | | |
|---|---|---|---|---|---|---|---|---|
| | CD-Ratio | | | | | MR-Steiger | | |
| $k$ | 10 SNPs | 9 SNPs | max | mean | min | max | mean | min |
| 1 | 0.021 | 0.022 | 0.004 | 4e-04 | 0 | 0.313 | 0.0417 | 0 |
| 2 | 0.033 | 0.029 | 0.015 | 0.0015 | 0 | 0.797 | 0.115 | 0 |
| 3 | 0.048 | 0.039 | 0.035 | 0.0043 | 0 | 0.958 | 0.1551 | 0 |
| 4 | 0.051 | 0.043 | 0.043 | 0.006 | 0 | 0.994 | 0.1818 | 0 |
| 5 | 0.05 | 0.048 | 0.047 | 0.006 | 0 | 0.999 | 0.1925 | 0 |
| $\beta_{YX} = 0.1$ | | | | | | | | |
| | CD-Ratio | | | | | MR-Steiger | | |
| $k$ | 10 SNPs | 9 SNPs | max | mean | min | max | mean | min |
| 1 | 0.024 | 0.024 | 0.004 | 4e-04 | 0 | 0.327 | 0.0441 | 0 |
| 2 | 0.066 | 0.075 | 0.023 | 0.0023 | 0 | 0.775 | 0.1131 | 0 |
| 3 | 0.112 | 0.127 | 0.056 | 0.0061 | 0 | 0.93 | 0.1511 | 0 |
| 4 | 0.133 | 0.12 | 0.085 | 0.0106 | 0 | 0.978 | 0.1801 | 0 |
| 5 | 0.134 | 0.134 | 0.112 | 0.0137 | 0 | 0.993 | 0.1892 | 0 |
| $\beta_{YX} = -0.1$ | | | | | | | | |
| | CD-Ratio | | | | | MR-Steiger | | |
| $k$ | 10 SNPs | 9 SNPs | max | mean | min | max | mean | min |
| 1 | 0.032 | 0.033 | 0.005 | 6e-04 | 0 | 0.306 | 0.0405 | 0 |
| 2 | 0.079 | 0.072 | 0.023 | 0.0024 | 0 | 0.756 | 0.1085 | 0 |
| 3 | 0.103 | 0.108 | 0.06 | 0.0067 | 0 | 0.938 | 0.155 | 0 |
| 4 | 0.153 | 0.161 | 0.086 | 0.0109 | 0 | 0.979 | 0.1755 | 0 |
| 5 | 0.153 | 0.151 | 0.108 | 0.0143 | 0 | 0.997 | 0.1905 | 0 |
| $\beta_{YX} = 0.2$ | | | | | | | | |
| | CD-Ratio | | | | | MR-Steiger | | |
| $k$ | 10 SNPs | 9 SNPs | max | mean | min | max | mean | min |
| 1 | 0.051 | 0.045 | 0.001 | 1e-04 | 0 | 0.292 | 0.0405 | 0 |
| 2 | 0.166 | 0.171 | 0.044 | 0.0046 | 0 | 0.693 | 0.1001 | 0 |
| 3 | 0.306 | 0.3 | 0.124 | 0.0133 | 0 | 0.843 | 0.1363 | 0 |
| 4 | 0.335 | 0.346 | 0.2 | 0.0233 | 0 | 0.926 | 0.1672 | 0 |
| 5 | 0.431 | 0.437 | 0.295 | 0.034 | 0 | 0.973 | 0.1819 | 0 |
| $\beta_{YX} = -0.2$ | | | | | | | | |
| | CD-Ratio | | | | | MR-Steiger | | |
| $k$ | 10 SNPs | 9 SNPs | max | mean | min | max | mean | min |
| 1 | 0.062 | 0.056 | 0.004 | 6e-04 | 0 | 0.264 | 0.0355 | 0 |
| 2 | 0.196 | 0.203 | 0.06 | 0.0063 | 0 | 0.646 | 0.0949 | 0 |
| 3 | 0.315 | 0.315 | 0.152 | 0.0159 | 0 | 0.844 | 0.1405 | 0 |
| 4 | 0.424 | 0.44 | 0.273 | 0.0311 | 0 | 0.926 | 0.1618 | 0 |
| 5 | 0.495 | 0.502 | 0.324 | 0.0392 | 0 | 0.976 | 0.1807 | 0 |

From Table A we can see that, when there was no causal effect, i.e. $\beta_{YX} = 0$, and when $k = 1$, i.e.

the sample sizes were small, CD-Ratio was a little conservative. As $k$ increased to 5, its empirical Type-I error rates would approach the nominal level 0.05. When there was a causal effect, i.e. $\beta_{YX} \neq 0$, as $k$ increased the power of CD-Ratio also increased, and the power with 10 or 9 SNPs was close and was higher than the maximum of using only single SNPs. For MR-Steiger, the maximum and mean relative frequencies from the 10 single SNPs gave some inflated Type-I error rates when $\beta_{YX} = 0$. Again it was because the SNPs had larger absolute correlations with $X$ than with $Y$, regardless of whether $X$ had a causal effect on $Y$ or not. Given its inflated type I error rates, the high power of MR-Steiger was not meaningful here. Note also here we considered only one possible causal direction of $X$ to $Y$.
