# Supplementary material for: Inferring causal direction between two traits in the presence of horizontal pleiotropy with GWAS summary data
Source: PLoS Genet. 2020 Nov 2;16(11):e1009105. doi: 10.1371/journal.pgen.1009105 (PMC7660933; doi:10.1371/journal.pgen.1009105)

S9 Text: Simulations to Study Selection Bias

Table A: Simulation results for estimating K_{YX} with no pleiotropy. The means, standard deviations (sd) and SEs (se) from each method are shown for each set-up based on 500 simulated datasets. μ_α represents the mean of the pleiotropic/direct effects (with standard deviation $\sigma_\alpha = 0$). N represents the number of top SNPs that are used.

No pleiotropy			CD-Ratio			CD-Egger			CD-GLS		
μ_α	K_{YX}	N	Mean($\hat{K}$)	sd($\hat{K}$)	Mean$(se(\hat{K}))$	Mean($\hat{K}$)	sd($\hat{K}$)	Mean$(se(\hat{K}))$	Mean($\hat{K}$)	sd($\hat{K}$)	Mean$(se(\hat{K}))$
0	0	10	-0.0001	0.0069	0.0052	0	0.007	0.0063	0	0.007	0.0063
		15	0.0001	0.0048	0.0042	0.0001	0.0048	0.0047	0.0001	0.0048	0.0047
		22	0.0001	0.0034	0.0035	0.0001	0.0034	0.0036	0.0001	0.0034	0.0036
0	0.2257	10	0.2261	0.0038	0.0041	0.226	0.0039	0.0042	0.226	0.0039	0.0043
		15	0.2261	0.0036	0.0038	0.2261	0.0036	0.0039	0.2261	0.0036	0.0039
		22	0.2256	0.0033	0.0036	0.2256	0.0033	0.0036	0.2256	0.0033	0.0036
0	-0.2344	10	-0.2346	0.0038	0.0042	-0.2346	0.0039	0.0042	-0.2345	0.0039	0.0043
		15	-0.2346	0.0035	0.0038	-0.2346	0.0035	0.0039	-0.2346	0.0035	0.0039
		22	-0.2341	0.0032	0.0036	-0.2341	0.0032	0.0036	-0.2341	0.0032	0.0036

Table B: Simulation results for estimating K_{YX} with balanced pleiotropy. The means, standard deviations (sd) and SEs (se) from each method are shown for each set-up based on 500 simulated datasets. μ_α represents the mean of the pleiotropic/direct effects (with standard deviation $\sigma_\alpha = 0.1$). N represents the number of top SNPs that are used.

Balanced pleiotropy			CD-Ratio			CD-Egger			CD-GLS		
μ_α	K_{YX}	N	Mean($\hat{K}$)	sd($\hat{K}$)	Mean$(se(\hat{K}))$	Mean($\hat{K}$)	sd($\hat{K}$)	Mean$(se(\hat{K}))$	Mean($\hat{K}$)	sd($\hat{K}$)	Mean$(se(\hat{K}))$
0	0	10	0.0007	0.1023	0.0053	-0.0057	0.0801	0.0741	-0.0056	0.0803	0.0741
		15	0.0013	0.0767	0.0044	-0.0017	0.0579	0.0547	-0.0017	0.0587	0.0551
		22	0.0013	0.0563	0.0037	-0.0007	0.0431	0.041	-0.0005	0.0436	0.0419
0	0.2257	10	0.2801	0.0615	0.0046	0.2531	0.0586	0.0591	0.2535	0.0587	0.059
		15	0.2479	0.0579	0.0041	0.2339	0.0508	0.0503	0.2341	0.051	0.0504
		22	0.2181	0.0531	0.0037	0.2201	0.0397	0.0393	0.2203	0.0402	0.0401
0	-0.2344	10	-0.2871	0.0636	0.0047	-0.265	0.0615	0.0628	-0.2652	0.0615	0.0627
		15	-0.2545	0.0595	0.0041	-0.2454	0.0524	0.0527	-0.2455	0.0525	0.0527
		22	-0.2233	0.0545	0.0037	-0.2296	0.0409	0.0408	-0.2295	0.0414	0.0416

Table C: Simulation results for estimating K_{YX} with directional pleiotropy. The means, standard deviations (sd) and SEs (se) from each method are shown for each set-up based on 500 simulated datasets. μ_α represents the mean of the pleiotropic/direct effects (with standard deviation $\sigma_\alpha = 0.1$). N represents the number of top SNPs that are used.

Directional pleiotropy											
			CD-Ratio			CD-Egger			CD-GLS		
μ_α	K_{YX}	N	Mean($\hat{K}$)	sd($\hat{K}$)	Mean$\left(se(\hat{K})\right)$	Mean($\hat{K}$)	sd($\hat{K}$)	Mean$\left(se(\hat{K})\right)$	Mean($\hat{K}$)	sd($\hat{K}$)	Mean$\left(se(\hat{K})\right)$
0.2	0	10	-0.0908	0.1345	0.0053	0.02	0.0529	0.0618	0.02	0.0529	0.0617
		15	-0.0347	0.0843	0.0044	-0.0017	0.0428	0.0508	-0.0017	0.0428	0.0507
		22	-0.0112	0.0538	0.0039	-0.0005	0.0386	0.0368	-0.0004	0.0392	0.0375
0.4	0	10	-0.1389	0.1189	0.0051	0.0297	0.041	0.086	0.0297	0.041	0.0859
		15	-0.0857	0.0824	0.0045	-0.0029	0.0381	0.0689	-0.0029	0.0381	0.0687
		22	-0.0183	0.0448	0.0041	-0.0004	0.0306	0.0291	-0.0003	0.0311	0.0297
0.6	0	10	-0.2007	0.1215	0.0052	0.0419	0.0363	0.0991	0.0418	0.0363	0.0989
		15	-0.1052	0.0673	0.0045	-0.009	0.032	0.0783	-0.009	0.032	0.0781
		22	-0.0317	0.0357	0.0042	-0.0003	0.024	0.0228	-0.0002	0.0244	0.0233
1	0	10	-0.2569	0.1313	0.0053	0.0553	0.0306	0.1098	0.0551	0.0306	0.1095
		15	-0.1162	0.0461	0.0045	-0.0106	0.0234	0.0854	-0.0108	0.0234	0.085
		22	-0.0506	0.0241	0.0043	-0.0002	0.0161	0.0153	-0.0002	0.0164	0.0156
0.2	0.2027	10	0.3346	0.1275	0.0055	0.0563	0.0751	0.0692	0.0563	0.075	0.0691
		15	0.2443	0.0844	0.0045	0.1211	0.0499	0.0519	0.1212	0.0499	0.0518
		22	0.1898	0.0514	0.0039	0.1987	0.0369	0.0355	0.1989	0.0375	0.0362
0.4	0.1614	10	0.1881	0.1075	0.005	0.0984	0.0587	0.0951	0.0983	0.0587	0.095
		15	0.1739	0.0857	0.0045	0.0996	0.0412	0.0698	0.0996	0.0412	0.0696
		22	0.1438	0.0448	0.0041	0.1593	0.0301	0.0285	0.1595	0.0307	0.029
0.6	0.1272	10	0.0887	0.1052	0.005	0.1067	0.0511	0.1023	0.1066	0.0512	0.102
		15	0.0851	0.0812	0.0044	0.0939	0.0342	0.0788	0.0938	0.0342	0.0786
		22	0.0944	0.0362	0.0042	0.1261	0.024	0.0225	0.1263	0.0244	0.023
1	0.0856	10	0.0104	0.1078	0.0051	0.1021	0.0307	0.1091	0.1019	0.0307	0.1089
		15	-0.0067	0.0553	0.0044	0.0636	0.0235	0.0869	0.0634	0.0235	0.0865
		22	0.0313	0.0244	0.0043	0.0851	0.0162	0.0152	0.0852	0.0165	0.0155
0.2	-0.2093	10	-0.4414	0.0938	0.0052	-0.1217	0.0642	0.0681	-0.1217	0.0642	0.0681
		15	-0.3054	0.0714	0.0046	-0.1886	0.0519	0.053	-0.1885	0.0519	0.0528
		22	-0.2172	0.0517	0.0039	-0.2061	0.037	0.0366	-0.206	0.0373	0.0373
0.4	-0.1649	10	-0.4235	0.0964	0.0053	-0.0929	0.046	0.0866	-0.093	0.046	0.0864
		15	-0.3038	0.0687	0.0048	-0.1428	0.04	0.0679	-0.1429	0.0399	0.0676
		22	-0.1796	0.0435	0.0041	-0.1636	0.0298	0.029	-0.1635	0.03	0.0296
0.6	-0.129	10	-0.4022	0.0538	0.0054	-0.0712	0.0367	0.1002	-0.0714	0.0368	0.0999
		15	-0.2979	0.0778	0.0048	-0.1129	0.032	0.0771	-0.113	0.032	0.0768
		22	-0.1552	0.0351	0.0042	-0.1285	0.0236	0.0228	-0.1285	0.0238	0.0233
1	-0.0862	10	-0.4157	0.055	0.0055	-0.0247	0.0261	0.1112	-0.0248	0.0261	0.1108
		15	-0.2396	0.0762	0.0047	-0.0882	0.0252	0.0843	-0.0883	0.0252	0.0839
		22	-0.1301	0.0238	0.0043	-0.0862	0.0159	0.0153	-0.0862	0.0161	0.0156

Figure A: Relative frequencies of decisions for causal direction of proposed methods when there is no causal relationship between X and Y ($K_{YX} = 0$) and no pleiotropic effects ($\mu_\alpha = 0, \sigma_\alpha = 0$) for different $N = 10, 15, 22$.

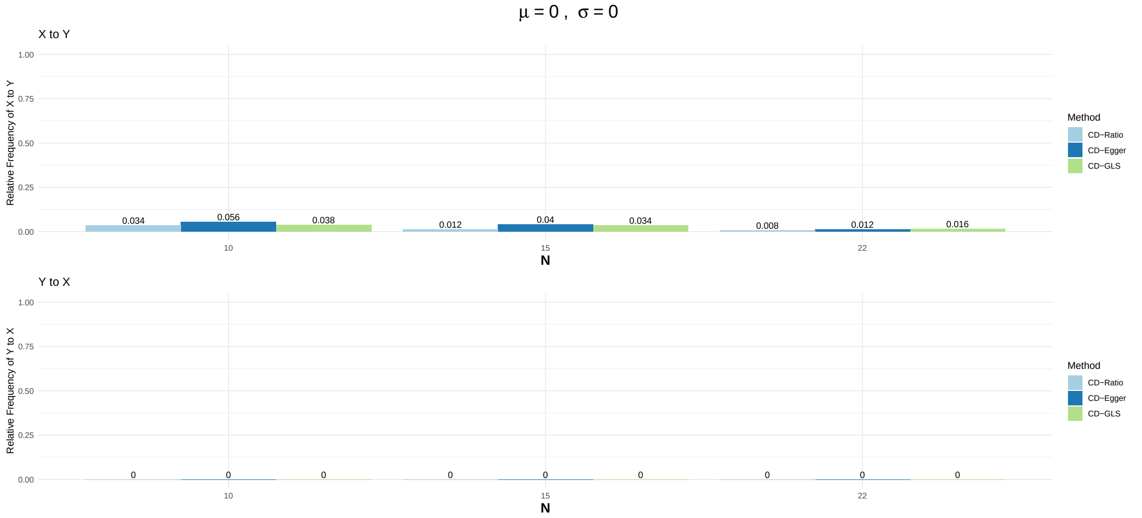

Figure B: Relative frequencies of decisions for causal direction of proposed methods when there is no causal relationship between X and Y ($K_{YX} = 0$) and balanced pleiotropic effects ($\mu_\alpha = 0, \sigma_\alpha = 0.1$) for different $N = 10, 15, 22$.

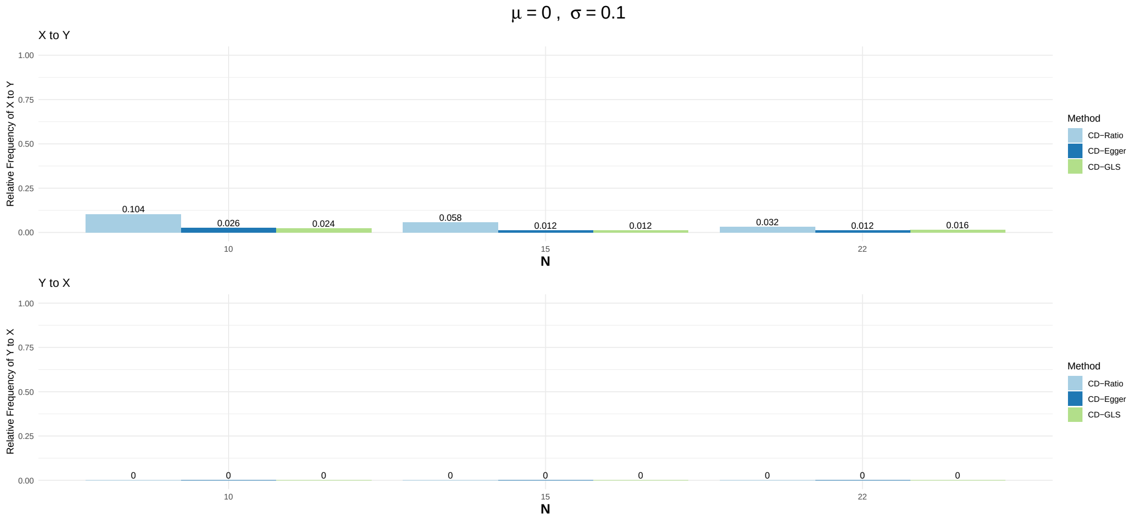

Figure C: Relative frequencies of decisions for causal direction of proposed methods when there is no causal relationship between X and Y ($K_{YX} = 0$) and directional pleiotropic effects ($\mu_\alpha = 0.2, \sigma_\alpha = 0.1$) for different $N = 10, 15, 22$.

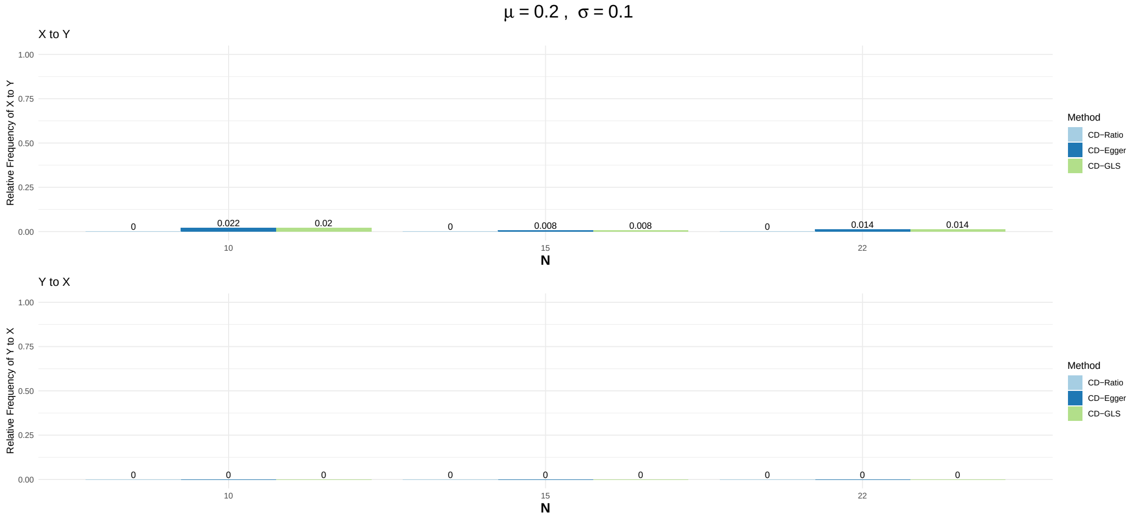

Figure D: Relative frequencies of decisions for causal direction of proposed methods when there is no causal relationship between X and Y ($K_{YX} = 0$) and directional pleiotropic effects ($\mu_\alpha = 0.4, \sigma_\alpha = 0.1$) for different $N = 10, 15, 22$.

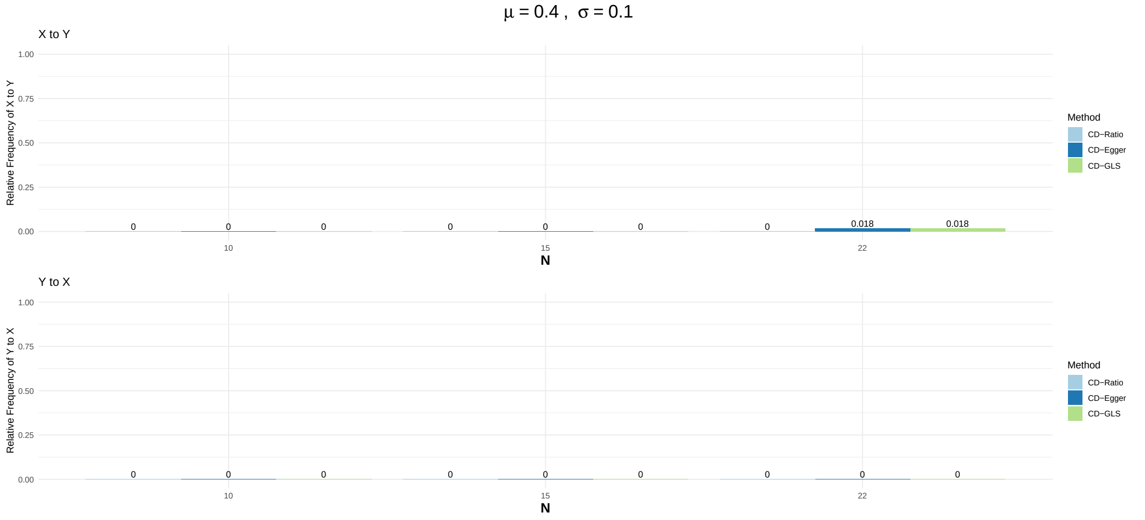

Figure E: Relative frequencies of decisions for causal direction of proposed methods when there is no causal relationship between X and Y ($K_{YX} = 0$) and directional pleiotropic effects ($\mu_\alpha = 0.6, \sigma_\alpha = 0.1$) for different $N = 10, 15, 22$.

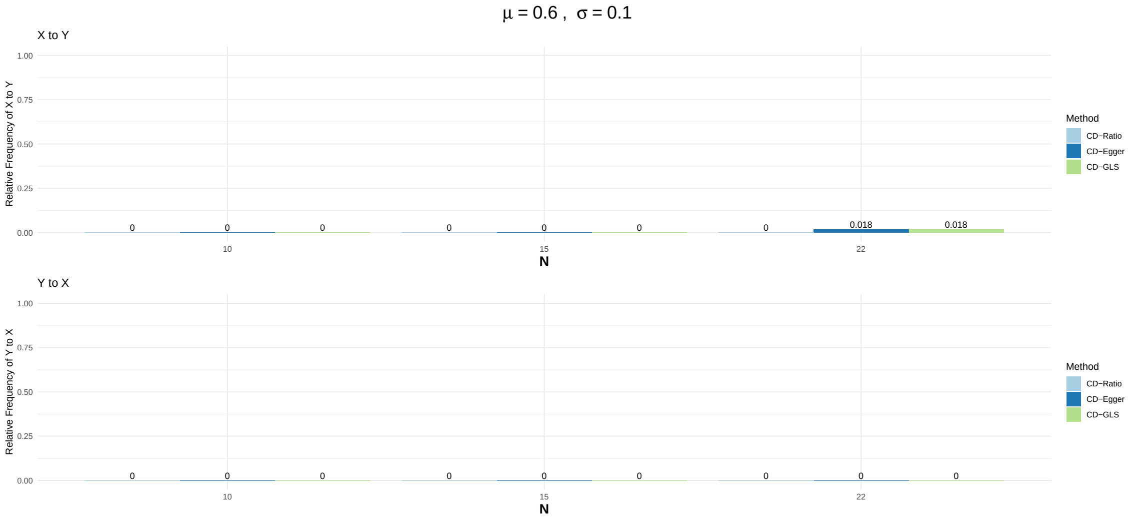

Figure F: Relative frequencies of decisions for causal direction of proposed methods when there is no causal relationship between X and Y ($K_{YX} = 0$) and directional pleiotropic effects ($\mu_\alpha = 1, \sigma_\alpha = 0.1$) for different $N = 10, 15, 22$.

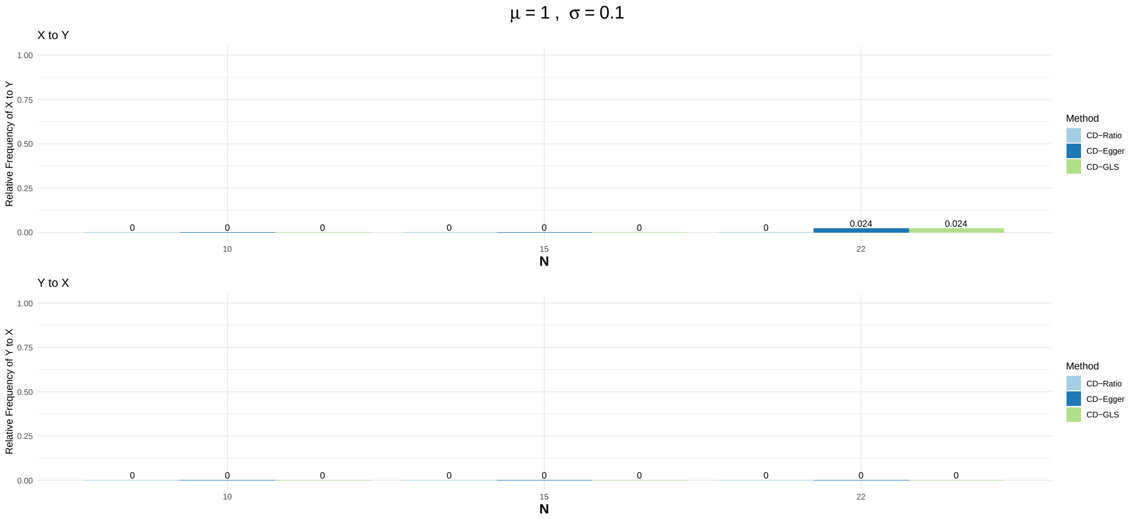

Supplement: S9 Text — (PDF) [file pgen.1009105.s009.pdf]
