# Supplementary material for: Inferring causal direction between two traits in the presence of horizontal pleiotropy with GWAS summary data
Source: PLoS Genet. 2020 Nov 2;16(11):e1009105. doi: 10.1371/journal.pgen.1009105 (PMC7660933; doi:10.1371/journal.pgen.1009105)

S10 Text: Fisher's Transformation to Absolute Correlations

In the left panel of Figure A, we draw the density function of normal distribution with mean 1 and standard error 1, i.e. $N(1,1)$, with blue color, and density function of its absolute value with red color. The right panel is for normal distribution with mean 1 and standard error 0.5, i.e. $N(1,0.5)$. We can see when standard error is 1, the original density function is different from the density function of absolute value. As standard error decreases to 0.5, the original density function is close to the density function of absolute value.

Figure A: Comparing original density function and density function of absolute value of normal distributions, left panel is for $N(1,1)$ and right panel is for $N(1,0.5)$.

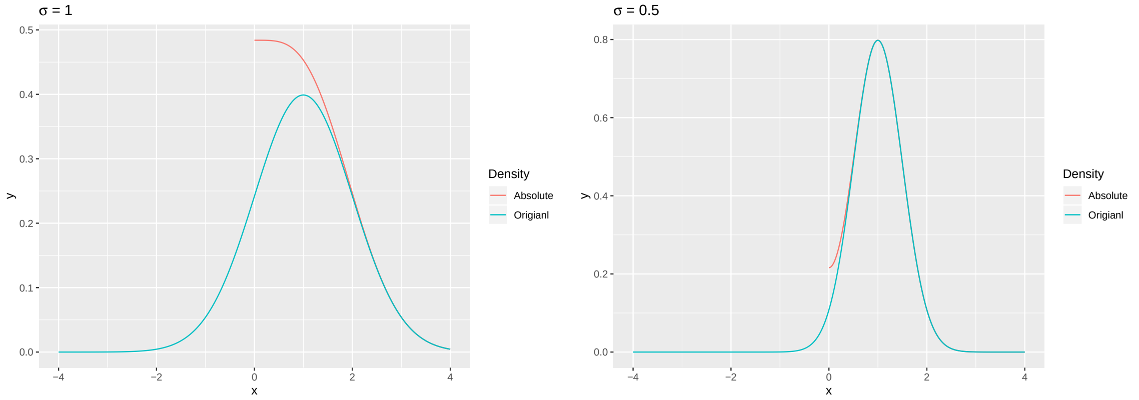

Supplement: S10 Text — (PDF) [file pgen.1009105.s010.pdf]
